# Using Personalized Avatars as an Adjunct to an Adult Weight Loss Management Program: Randomized Controlled Feasibility Study

**DOI:** 10.2196/36275

**Published:** 2022-10-05

**Authors:** Maria Horne, Maryann Hardy, Trevor Murrells, Hassan Ugail, Andrew John Hill

**Affiliations:** 1 Faculty of Medicine and Health School of Healthcare University of Leeds Leeds United Kingdom; 2 Faculty of Health Studies School of Allied Health Professions and Midwifery University of Bradford Bradford United Kingdom; 3 Florence Nightingale Faculty of Nursing, Midwifery and Palliative Care King's College London London United Kingdom; 4 University of Bradford Centre for Visual Computing Bradford United Kingdom; 5 Institute of Health Sciences School of Medicine University of Leeds Leeds United Kingdom

**Keywords:** avatar, feasibility, obesity, weight loss, weight management

## Abstract

**Background:**

Obesity is a global public health concern. Interventions rely predominantly on managing dietary intake and increasing physical activity; however, sustained adherence to behavioral regimens is often poor. The lack of sustained motivation, self-efficacy, and poor adherence to behavioral regimens are recognized barriers to successful weight loss. Avatar-based interventions achieve better patient outcomes in the management of chronic conditions by promoting more active engagement. Virtual representations of *self* can affect real-world behavior, acting as a catalyst for sustained weight loss behavior.

**Objective:**

We evaluated whether a personalized avatar, offered as an adjunct to an established weight loss program, can increase participant motivation, sustain engagement, optimize service delivery, and improve participant health outcomes.

**Methods:**

A feasibility randomized design was used to determine the case for future development and evaluation of avatar-based technology in a randomized controlled trial. Participants were recruited from general practitioner referrals to a 12-week National Health Service weight improvement program. The main outcome measure was weight loss. Secondary outcome measures were quality-of-life and self-efficacy. Quantitative data were subjected to descriptive statistical tests and exploratory comparison between intervention and control arms. Feasibility and acceptability were assessed through interviews and analyzed using framework approach. Health Research Authority ethics approval was granted.

**Results:**

Overall, 10 men (n=7, 70% for routine care and avatar and n=3, 30% for routine care) and 33 women (n=23, 70% for intervention and n=10, 30% for routine care) were recruited. Participants’ initial mean weight was greater in the intervention arm than in the routine care arm (126.3 kg vs 122.9 kg); pattern of weight loss was similar across both arms of the study in T0 to T1 period but accelerated in T1 to T2 period for intervention participants, suggesting that access to the self-resembling avatar may promote greater engagement with weight loss initiatives in the short-to-medium term. Mean change in participants’ weight from T0 to T2 was 4.5 kg (95% CI 2.7-6.3) in the routine care arm and 5.3 kg (95% CI 3.9-6.8) in the intervention arm. Quality-of-life and self-efficacy measures demonstrated greater improvement in the intervention arm at both T1 (105.5 for routine care arm and 99.7 for intervention arm) and T2 (100.1 for routine care arm and 81.2 for intervention arm). Overall, 13 participants (n=11, 85% women and n=2, 15% men) and two health care professionals were interviewed about their experience of using the avatar program.

**Conclusions:**

Participants found using the personalized avatar acceptable, and feedback reiterated that seeing a future *self* helped to reinforce motivation to change behavior. This feasibility study demonstrated that avatar-based technology may successfully promote engagement and motivation in weight loss programs, enabling participants to achieve greater weight loss gains and build self-confidence.

**Trial Registration:**

ISRCTN Registry 17953876; https://doi.org/10.1186/ISRCTN17953876

## Introduction

### Background

Obesity is a global health concern [1] and a health priority in the United Kingdom [2,3]. It is associated with a range of increased health risks including diabetes (type II), heart disease, cancer, depression, and mental health issues [4], affecting societal engagement and quality of life [5]. It has also been identified as a mortality and morbidity risk factor for COVID-19 [6]. In addition to affecting the health of individuals, obesity increases demand for, and complexity of, health care and is estimated to cost the UK National Health Service (NHS) £5 billion (US $5.739 billion) annually with a projected increase of £1.9 billion to £2 billion (US $2.08 billion to US $2.19 billion) per annum by 2030 without intervention [7]. Consequently, in terms of individual, societal, and economic well-being and sustainability, developing effective interventions to reduce population obesity is imperative.

Obesity interventions in adults rely predominantly on managing dietary intake and increasing physical activity [8]. Systematic reviews have evaluated the effectiveness of interventions, and multicomponent interventions have been acknowledged to be more effective for weight loss than single-component approaches [9,10]. Common barriers to successful weight loss among adults with obesity have been identified as lack of sustained motivation and poor adherence to behavioral regimens [11,12]. However, interest in the use of digital technologies to support health behavior change is growing [13,14].

Technology-based interventions for weight loss have shown promise in the short term [15,16]; however, the potential of computer engineering has not been fully realized when designing weight loss interventions [17]. It has been argued that new multicomponent (integrated) electronic platforms integrating education with individually tailored weight loss programs, including the promotion of autonomous motivation, self-efficacy, self-regulation, and positive body image, may present a way forward for wide-scale weight loss solutions and obesity management [18-20]. An area that has significant potential to be exploited as a catalyst for weight loss behavior modification within health care is virtual reality (VR) and the creation of personal avatars [15]. VR enables people to experience an alternate visual reality, often through an avatar (computerized representation of self). Avatar technology is well established within the computer gaming industry, and third-person perspective of self within a VR setting has been shown to promote emotional engagement [21,22].

On the basis of the social cognitive theory by Bandura [23] (behavior learning through observation of models) and self-perception theory by Bem [24] (inference of own attitudes by observing self from a third-party perspective), studies have demonstrated a link between virtual representations of self and real-world behavior and attitudes, which is considered as a consequence of individuals identifying with their avatar [25,26], particularly when the avatar is self-resembling [27], and experience of presence within the computer-mediated environment [28].

The potential of VR as an intervention has been explored across a spectrum of health and well-being conditions [25,26,29,30], and it has been recommended as a potential behavior modification tool for tackling obesity [31,32]. Studies have shown that within the experimental environment, an individual’s behavior conforms to their digital self-representation (Proteus effect) [33-35]. Importantly, observing a self-resembling avatar modeling an activity (eg, exercise) within a VR environment, as opposed to a generic avatar, differentially positively influences real-world behavior, thus increasing actual engagement with the activity [29,36]. This connectedness between the actual and digital self has been further explored using aging algorithms to present individuals with personalized virtual futures influenced by choices made today [37,38], and the results suggest that observation of the future self within a web-based environment may vicariously reinforce today’s desirable behaviors and attitudes.

Transforming the appearance of self through an avatar is a particularly powerful motivational application, but there has been limited translation of this technology into health care service delivery. So far, no identified study has directly applied the technology to the clinical setting as an adjunct to an existing obesity intervention or overtly included people with obesity within the study sampling frame.

### Aims

We proposed to develop a self-resembling avatar generation program to allow individuals to explore potential health futures related to obesity, including observing their self-avatar gain and lose weight and information on the associated health risks at BMI boundaries. This feasibility study tested the avatar design capabilities; clinical application; and underlying premise that this technology, provided as an adjunct to an existing weight loss program, will positively influence participant motivation.

## Methods

### Study Design

This was a 2-phase, sequential, mixed methods feasibility study, incorporating a feasibility randomized controlled trial (RCT) with a subsequent qualitative component, to determine the case for, and the parameters of, a future RCT. CONSORT-EHEALTH (Consolidated Standards of Reporting Trials of Electronic and Mobile Health Applications and Online Telehealth) guidelines were used to report the study (Multimedia Appendix 1).

### Ethics Approval

Ethics approval for the study was obtained from the University of Bradford and Health Research Authority (research ethics committee reference 18/NE/0286), and it was registered with the International Standard Randomized Control Trial Number (17953876). All participants were screened for suitability, by a registered medical clinician specializing in obesity, and psychological vulnerability. All participants provided written informed consent, and a process of medical referral and reporting of any potential harm as a result of participation in the study was established.

### Participants

Participants were patients with obesity or those who were overweight from within West Yorkshire, England, referred to the Mid Yorkshire Hospitals NHS Trust weight improvement service (March 2019 to December 2019). Inclusion criteria were the following: (1) adults aged 18 to 65 years, (2) BMI >30 kg/m^2^, (3) referred from GP, and (4) no known comorbidities or medical treatment that may influence dietary intake or weight loss achievement (eg, type I diabetes or current medication with weight gain as a known side effect).

Exclusion criteria were the following: (1) pregnant women, (2) children, (3) older individuals (aged >65 years), (4) BMI >45 kg/m^2^, and (5) people considered to be psychologically vulnerable.

A parallel group approach was undertaken with participants assigned to routine weight management program (routine care) or routine weight management program and avatar (intervention) and treated according to group assignment.

### Recruitment

Potential participants were identified by the weight management service clinical team (Mid Yorkshire Hospitals NHS Trust) following initial assessment for suitability to participate in a weight management program and study participation screening. Potential participants were given an information pack, including invitation letter; information sheet; permission-to-contact form; and postage-paid envelope to read, complete, and return after the appointment. The forms were returned to the weight management service, and a secure database of potential participants was created by the NHS research administrator assigned to this project. The researcher confirmed with the administrator on a weekly basis about those patients who had returned the permission-to-contact forms and telephoned those patients interested in participating to describe the study further and confirm their willingness to participate. A cluster randomization approach was adopted to allocate patients to routine care or routine care and avatar based on the geographic location of the weight management service intervention.

A subsample of participants within both arms who completed the 12-week weight management program were invited to participate in individual interviews, 3 to 6 months after commencement of the intervention, and share their experiences of using the avatar program.

Health care professionals (HCPs) involved in delivering the weight loss program and who had experience of using the avatar program were invited to participate in a semistructured interview at the conclusion of the study’s data collection phase. This was done to explore their experiences of using the avatar program as an adjunct to routine care.

### Intervention

The avatar VR program (creation and display) was developed through an iterative design process with service users and HCPs. Service user volunteers were recruited from the University of Bradford service user group, and HCP participants were recruited from the Mid Yorkshire Hospitals NHS Trust weight management service. Feedback at each stage of development ensured that the final avatar program design was optimized before commencing the feasibility study.

The definition of avatar adopted for this study was a web-based 3D entity whose appearance and body proportions resembled that of the user. In this sense, the avatar was a close representation or a *digital clone* of the individual, allowing the individual to relate to it in terms of appearance and body proportions.

The avatar was developed using standard 3D modeling technology. Standard human 3D shapes were developed using Maya 3D modeling software (Autodesk). This 3D model is flexible, permitting body parts (eg, size of the lower abdomen) to be changed using simple numbers or parameters. Therefore, the parameterized 3D model can be efficiently manipulated using a handful of parameter values determined by physical measurements and photographs of the individual acquired using a tablet device (iPad).

The weight management routine care program operated over 12 weeks. Patients who were allocated to receive the intervention (routine care and avatar) attended the weight improvement service for their initial appointment (T0). Additional time was allocated for this appointment to meet the researcher and for baseline anthropomorphic measurements and digital photographs to be acquired. These parameters were inputted into the avatar creation program while participants completed three questionnaires—(1) Impact of Weight on Quality of Life-lite [39], (2) Weight Efficacy Lifestyle-Short Form questionnaire [40], and (3) EQ-5D-5L [41]—to monitor changes in well-being during the study period and identify the best tool to be adopted in a future RCT, if warranted. Once the avatar was generated (<5 minutes after data input), the participant was introduced to their avatar and program operation and given a unique username and password to allow them to access their avatar on the web via computer or tablet. Participants were encouraged to explore how increasing or decreasing weight affects avatar appearance, considering the 5% and 10% weight loss goals promoted by the weight management routine care program alongside the BMI boundary health risks as their avatar increased and decreased in weight.

Follow-up appointments with the researcher coincided with weight management service routine care appointments at 1 month (T1; immediate effect) and 3 months (T2; short-term effect after program completion). A further appointment to meet with the researcher was planned for 6 months (T3; 3 months after program completion—moderate-term progress). At each of these appointments, the researcher collected the completed surveys and inputted any change in weight into the avatar program. The changes in avatar appearance were presented and discussed with the participants at each researcher appointment, and participants were encouraged to use the avatar to visualize progress.

### Routine Care

Routine care was the standard 12-week weight management program without the provision of an avatar. Participants allocated to receive routine care were provided the same number of appointments with the weight improvement service as those allocated to the intervention group and met the researcher to complete questionnaires and acquire body measurements, photographs, and weight change data at the same points as those participating in the intervention arm. However, although routine care participants underwent the same process of meeting the researcher and replicating the activity of the intervention, visualization of, and access to, their avatar was withheld until the final weight management service appointment (12 weeks).

### Outcome Measures

The primary outcome measure was weight loss based on body weight (kg) and calculated BMI (kg/m^2^). Assessment of weight loss was planned to be conducted at baseline and after 3 months and 6 months.

The secondary outcome measures were uptake and continuation rates and changes in self-perceived quality of life and self-efficacy.

### Sample Size

The target sample size for the feasibility study trial was between 30 and 60 patients, overall [42]. The sample size for interviews with service users and HCPs was based on data saturation, with 10 to 16 interviews anticipated [43].

### Data Analysis

Data analysis focused on describing the key feasibility outcomes using descriptive statistical tests, including calculation of means and frequencies. Mean (SD and 95% CI) and median (IQR) values were reported by the study arm (routine care and routine care and avatar) for the primary outcomes of weight (kg) and BMI and secondary effectiveness outcome data collected through the questionnaires. A generalized linear repeated measures mixed effects model for weight (kg) was used to account for the discrete timing of the follow-up assessments and adjust for baseline measures (T0). The aim of that model was to obtain an estimate of variability (residual SD) for weight change and to estimate the mean change in weight (95% CI) from baseline (T0) to T1 and T2, which will then be used to inform the sample size calculation for a future definitive trial, if warranted.

Qualitative data were analyzed using framework approach [44]. The first author coded all the transcripts. Then, the codes were discussed with the research team, who together developed the indexing scheme that was used by the first author to chart data. These charts were shared with others in the team to explore and interpret the data together and decide the final themes. Consensus on themes and subthemes was reached through discussion.

## Results

### Recruitment

The number of potential participants who met the recruitment criteria was less than that expected. This was mainly owing to the greater than expected proportion of referrals to the weight management service related to people with BMI >45 kg/m^2^. In addition, restrictions and changes in service delivery from face-to-face to telephone appointments as a result of the COVID-19 pandemic influenced the final stages of data collection. Therefore, T3 (6 months) data were not collected for most participants, and analysis was restricted to T0 to T2.

**Figure 1:**
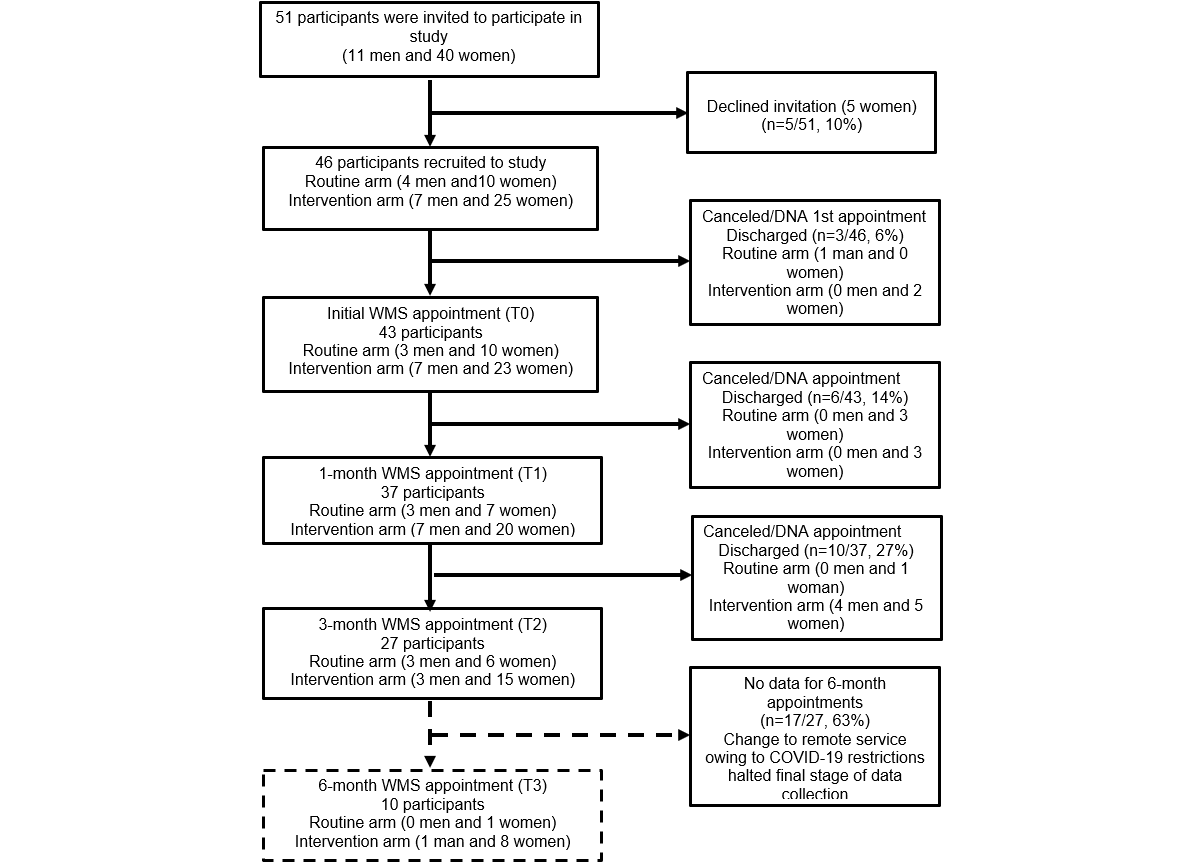
Participant flowchart. DNA: did not attend; WMS: weight management service.

Recruitment was conducted between March 2019 and December 2019. During this time, 51 potential participants were identified as being suitable for participation in the study, and information about the study was provided. Of these 51 participants, 5 (10%) declined participation and 3 (6%) did not attend the initial appointment and were discharged from the weight management service (Figure 1).

### Participant Characteristics

Baseline data were collected for 84% (43/51) of the participants. A total of 10 men were recruited into the study (n=7, 70% into the routine care and avatar arm and n=3, 30% into the routine care arm). At the beginning of the program, their mean age was 50 years 10 months (range 39-63 years) and mean weight was 140.2 (range 114.6-173.2) kg. A total of 33 women were enrolled into the study at T0 (n=23, 70% into the intervention arm and n=10, 30% into the routine care arm). At the beginning of the program, their mean age was 40 years 1 month (range 21-57 years) and mean weight was 120.5 (range 89.6-156.4) kg. Mean BMI was 43.3 (range 38.6 to 49.5) kg/m^2^.

### Weight

**Figure 2:**
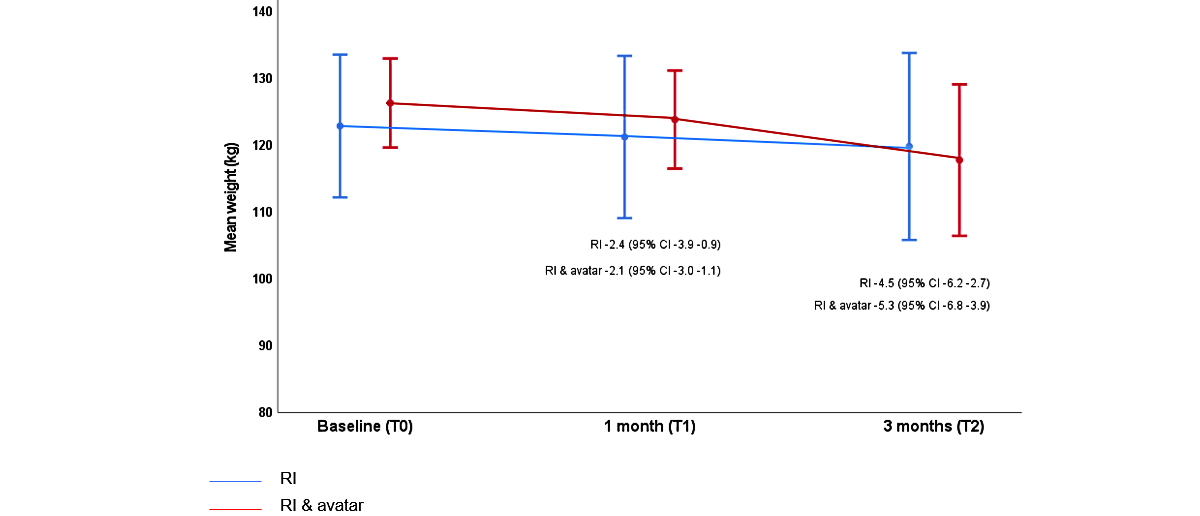
Weight (kg) by visit (cross-sectional means and SDs). Note: mean person-level change from baseline (95% CI) is shown for both arms at 1 month and 3 months. RI: routine intervention.

The initial mean weight of participants was greater in the intervention arm than in the routine care arm (126.3 kg vs 122.9 kg), but the pattern of weight loss was similar across both arms of the study in the T0 to T1 period. During T1 to T2, weight loss accelerated in the intervention arm, suggesting that access to the self-resembling avatar may promote great engagement with weight loss initiatives in the short to medium term (Figure 2).

The mean change in weight of the participants from T0 to T2 was 4.5 kg (95% CI 2.7-6.3) in the routine care arm and 5.3 kg (95% CI 3.9-6.8) in the intervention arm. The initial mean BMI of participants was greater in the intervention arm than in the routine care arm (44 kg/m^2^ vs 42.4 kg/m^2^). However, reflecting the great weight loss in the intervention arm, the mean BMI of participants in both groups was identical at T2 (BMI 41.1 kg/m^2^).

### Quality of Life and Self-efficacy

**Figure 3:**
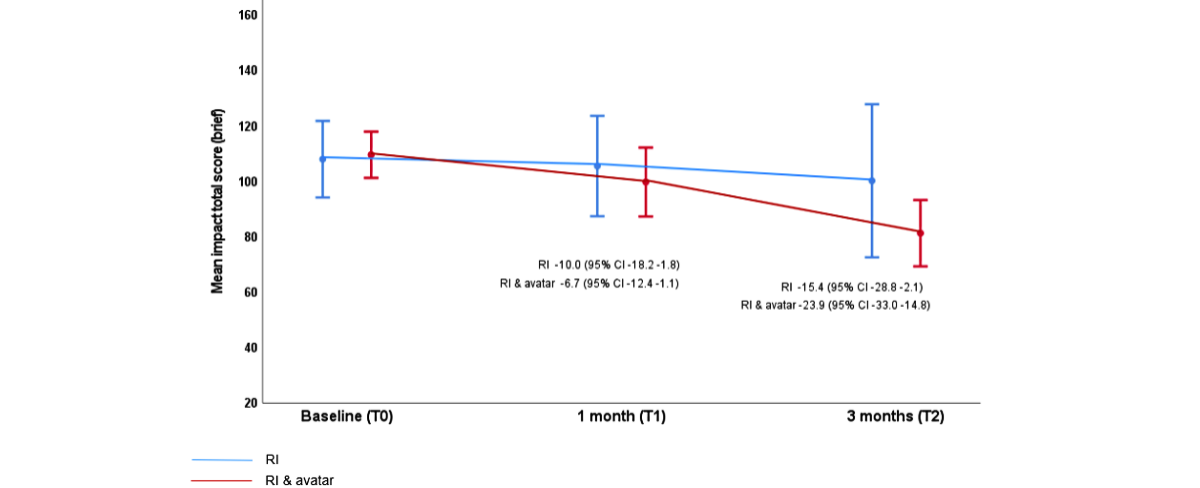
Impact of Weight on Quality of Life-lite total score (brief) by visit (cross-sectional means and SDs). Note: mean person-level change from baseline (95% CI) is shown for both arms at 1 month and 3 months. RI: routine intervention.

Quality of life and self-efficacy showed improvements, as seen in participants’ scores across both arms of the study, but greater improvement was noted within the intervention arm (Figure 3).

**Figure 4:**
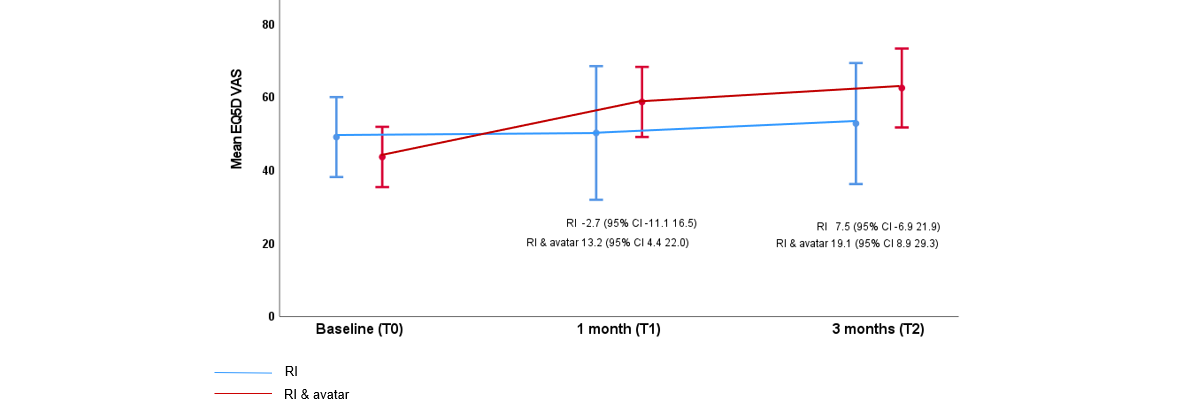
EQ5D VAS by visit (cross-sectional means/SDs). Note: mean person-level change from baseline (95% CI) is shown for both arms at 1 month and 3 months. RI: routine intervention.

Mean initial scores were comparable across the arms (107.9 in the routine care arm and 109.5 in the intervention arm), but participants in the intervention arm reported greater improvement at both T1 (105.5 in the routine care arm and 99.7 in the intervention arm) and T2 (100.1 in the routine care arm and 81.2 in the intervention arm). Improvement in participants’ self-assessment of health at the time of appointment was also noted over the study time frame for participants in both arms of the study using EQ-5D-5L Visual Analog Scale (VAS) score; however, once again, great improvement was noted within the intervention arm. Mean VAS scores for the routine arm were 49.1 (T0), 50.2 (T1), and 52.8 (T2), whereas for the intervention arm, mean VAS scores were 43.6 (T0), 58.7 (T1), and 62.5 (T2, Figure 4.

Having access to and visualizing changes in the self-resembling avatar improved weight loss motivation and perception of self–well-being. In contrast, the Weight Efficacy Lifestyle Questionnaire identified great improvements in participant self-belief in controlling eating behaviors in the routine care arm, with mean improvement in score between T0 and T2 of 17.6 in the routine arm and 12.1 in the intervention arm. It is unclear why the results from this questionnaire contrast with the findings of the other assessments. This may reflect the wording of statements, purpose of questionnaire, or interpretation by participants. However, it is important to note that the use of a single questionnaire alone will not allow for understanding of the complex motivations, enablers of, and barriers to weight loss.

### Experiences of Using a Web-Based Avatar

In total, 13 participants (n=11, 85% women and n=2, 15% men) and two HCPs volunteered to be interviewed about their experiences of using a web-based avatar on a weight loss program. Regarding acceptability, most participants found using the personalized avatar as an acceptable and positive experience:

Oh, I think the avatar’s brilliant. It’s really good to be able to see where you were and then to slowly see the progress because you can’t always see it when you are looking in the mirror...I get comments; people go have you lost some weight? And I’m like oh yeah just a bit, but I can’t see that I have lost weight but when you look at that [Avatar] you can see that even just a bit of weight makes that much difference.

MF45; female participant

However, a few participants found the initial viewing of the avatar as uncomfortable, but reported that the appearance of their avatar was a “reality check” of their current selves, which provided the added motivation to lose weight:

...It was all a bit shocking to be fair [seeing avatar for first time] ...I don’t look at myself very much in the mirror...

MM47; male participant

It gives you an objective look on how others would see you.

MF13; female participant

Devastated - that I had let myself get like that so...yeah...embarrassed...it spurred me on to get rid of all that round my belly...I know...as I get thinner I will get fitter and get rid of this lung issue and then I will get to where I want to be and I will go to the gym more and even do more at home. I will definitely walk the dog more; I’m trying to walk the dog faster so I am exerting myself more...so yeah, seeing that end result is brilliant to [compare with] where I was.

MF23; female participant

Visual perception of the personalized avatar assisted some participants to visualize a potential future reality:

It helps me to visualize what I could, will be...what I am going to look like.

MF17; female participant

Participants also reported added psychological benefits, such as increased motivation and improved self-esteem and self-confidence:

I need the visual to keep me motivated.

MF22; female participant

Helps your self-esteem...when you can see it rather than someone just telling you.

MF39; female participant

I feel like I am better equipped to kind of like be in control of myself.

MF02; female participant

Participants reported no concerns regarding the questionnaires used, despite some questions being personal and related to physical intimacy:

There are some personal questions talking about intimacy and things, its important though because it is a part...you know that is...when you put weight on you feel undesirable especially when your partner is thin as a rake and...you weren’t, you looked completely different when you met and you have been together you know...donkeys years you know, it is a big part of it, but I think it is important, you have to highlight every area or else you won’t address your issues will?

MF22; female participant

Another participant reported that the questionnaires were good at pinpointing the physical and emotional issues that people with obesity have. Another participant talked about the positive boost received when reflecting back on the scores from T0 to T2 and seeing positive change in the scores that further embedded the feeling of well-being and achievement:

...I think they pinpointed all the physical and emotional issues that people who are overweight probably face or are worried about. So yes, it was good and it was good that I could give honest answers and really think about each question.

MF01; female participant

Both health care practitioners were dieticians or nutritionists, and both felt that the personalized avatar, as an adjunct to a weight management program, was a useful addition to the questionnaires available:

I think it exceeded my expectations...because obviously she takes a photo of the patient and then it’s got their face on it and obviously their body...I know it didn’t have any clothing or anything like that [underwear only], but I don’t think that really matters because they get to see the actual body without the clothing...I think that’s better because they can see their skin and where they are actually carrying the weight...I think overall it has been beneficial to have it as part of the program.

HCP1; female HCP

I don’t think I expected so much emotion, not the extent where people needed a lot of comforting...I didn’t expect that no...Um...but I think it was a positive thing generally, it was something people could use to kind of scale down...It massively helped...just by dropping the BMI by a little bit or moving the scale option to do that...You know I would always highlight that this wasn’t just about aesthetics although, you know everyone kind of wants to be a smaller dress size, but it’s not really about that, it’s about your health. It was quite good to drop it and show if your BMI was lowered by this much, your risk of diabetes reduces by this much and I think that’s when it kind of hits people because then they are working to more health-related goals.

HCP2; female HCP

The addition of a personalized avatar to the standard NHS program was reported as helpful in terms of patients being able to visualize *reality* and confront denial:

I feel like the avatar has been a great help for the patients that were on the MotiVar study...a lot of the patients were able to see what they actually look like, because having worked in the service for a few years now...I do understand a lot of the patients don’t look in the mirror for example or they tend to be in denial about their weight or...they might be confident to lose the weight but then they know they are going to struggle to keep it off and that tends to knock them back if they have done several diets through their lifetime...but I think seeing that reality where you know they get to see the avatar and with their face on it as well...where they don’t have to look in the mirror but they see themselves on the screen. I think it is a reality check for a lot of them, but also think it’s a lot more real?

HCP1; female HCP

I felt like with the females...because they could visualize it...it became something a bit more real and people made comments that existing apps they had used weren’t realistic...one that did actually speak about that and she had downloaded a few apps in the past, but it wasn’t like, proportionate to her and you didn’t have to take so many measurements it was just your height.

HCP2; female HCP

Both HCPs felt that, for most participants, visualizing the personalized avatar assisted with the motivation to take up and maintain the weight loss program and to visualize personal goals:

...When they are starting something like this program, I think it (personalized avatar) definitely helps in terms of motivation and giving them that kick start that they need, but also it helps to visualize their goal as well because of what the avatar does offer...how to select the different weights and seeing what the goal weight is but again you can check what your 5% target is and then see the difference as well when the weight does change on the avatar. I think it’s really good to see it.

HCP1; female HCP

Health care practitioners reported no difficulties in administering the questionnaires and felt that they were appropriate for assessing confidence during the weight management program.

## Discussion

### Principal Findings

This study aimed to investigate the feasibility, acceptability, and implementation of a randomized design to determine the case for future development and evaluation of avatar-based technology in an RCT.

Following the recommendations for feasibility studies [45], feasibility was assessed by examining recruitment and retention (attrition) rates, together with participants’ and HCPs’ experiences of the use of avatar-based technology as an adjunct to a weight management program. In addition, we reported the pre-post intervention effect sizes on primary (weight loss) and other outcomes.

Recruitment was found to be feasible, with more than half of the contacted people consenting to participate, and the target of recruiting at least 30 participants into the intervention arm during the 6-month recruitment period was met. Reasons for declining to participate in the study were disinterest in the study and lack of guarantee of being in the intervention arm. Overall, 7% (3/46) of the recruited participants failed to attend their initial appointment and were discharged from the weight management service. The number of individuals recruited into the intervention arm (30/43, 70%) suggested that a personalized avatar, as an adjunct to a weight management program, will be feasible to offer and will be of interest to participants. However, attrition from the program was relatively high (19/46, 41%).

Regarding acceptability, all study participants mentioned experiencing benefits from using the avatar-based technology. Most participants emphasized the importance of the avatar’s appearance as a reality check, whereas HCPs felt that it was important for patients to visualize *reality* and confront denial.

Most participants in our study also reported added psychological benefits, such as increased motivation to lose weight and improved self-esteem and self-confidence.

Finally, although the feasibility study was not powered to detect a difference in weight, the study found 10% greater mean weight loss with routine care and avatar compared with routine care weight management program.

### Comparison With Previous Studies

Attrition and nonadherence are known to be common problems across all weight loss interventions, with reported mean attrition rates ranging from 10% to >80% [46-49]. Therefore, further studies are required to determine the role that avatar-based technology may have in reducing attrition and supporting engagement within a weight management intervention.

The importance of the avatar’s appearance in terms of visualizing *reality* and confronting denial builds on, and is consistent with, studies suggesting that mental imagery can induce strong positive and negative effects [50,51]. For example, Ridgway and King [51] found that when participants viewed their 3D avatars, their overall body satisfaction and mood decreased compared with that observed after viewing the baseline reports. However, participants also reported wanting to engage in greater appearance management behaviors after viewing the avatar compared with their baseline reports, suggesting that the avatar stimulated a change in perception of self and behavior. Similarly, Park [50], investigating how body image discrepancy or body satisfaction leverages behavioral intention for weight regulation among nonclinical participants who were aged ≥18 years, found that after participating in a web-based avatar session, those who demonstrated high body dissatisfaction exhibited great intention to be involved in behavioral change to achieve healthy body weight. In contrast, increased body image discrepancy in itself did not trigger an intention to lose weight after web-based avatar experience [50], confirming the multifactorial nature of self-perception and motivation to change behavior.

Weight management services are generally poorly adhered to [48], perhaps as a consequence of high body dissatisfaction, stigma, and discrimination toward people who are obese [52]. Viewing one’s anthropometric web-based avatar can affect the viewer’s self-body perception through comparative evaluation of self-concepts and lead to self-acceptance [53]. The initial discomfort experienced by some study participants on viewing their avatar appeared to provide the trigger needed for some to motivate them to change their behavior and adhere to a weight management program and warrants further investigation.

Our findings around the added psychological benefits, for example, increased motivation to lose weight and improved self-esteem and self-confidence, builds on and is consistent with previous studies suggesting that the experience of using an avatar can increase self-efficacy [54-56]. However, the literature suggests that this experience does not consistently translate into real-world settings and target health behaviors [56], thus supporting the need for further studies in this area.

Weight loss was similar between T0 to T1 for both routine care and avatar arm and routine care arm. However, from T1 to T2, weight loss was greater in the routine care and avatar arm, suggesting that high level of motivation was maintained. The reason for this was not explored directly, but can be explained by the placebo effect of participating in a research trial [57]. Participants in both arms knew that they were part of a research study and were meeting researchers and the weight management team; this may have had an equal impact on diet motivation [57].

A recent systematic review [15], which focused on the inclusion of avatar technology in weight loss interventions, identified greater weight loss and weight maintenance compared with routine interventions. However, the differences were not consistent in terms of statistical or clinical significance [15]. No full-powered trial has been undertaken, and, therefore, although the findings in terms of weight loss demonstrate potential, large studies are required to confirm the results.

### Limitations

The feasibility trial design had several strengths, and, importantly, this was the first study of its type, conducted in a health care setting with a GP referral weight management service. As this was a feasibility study, assessing the effectiveness of the intervention was not the primary aim. However, future studies should examine the effectiveness of the avatar within an RCT design and better understand the economic impact.

Limitations of this study include the small sample size and relatively high attrition. However, it still remains as one of the larger sample size studies reported so far. Despite these limitations, this feasibility study has illustrated that avatar-based technology may successfully promote engagement with and motivation to lose weight as part of a weight management program.

This feasibility study demonstrates the possibilities of using avatar-based technology to motivate engagement with a weight management program in the short term. The findings also suggest that avatar-based technology may support greater self-confidence, belief, and efficacy in weight loss ambitions.

### Conclusions

We investigated the feasibility, acceptability, and implementation of a randomized design to determine the case for future development and evaluation of avatar-based technology in an RCT. This feasibility study demonstrates the possibilities of using avatar-based technology to motivate engagement with a weight management program in the short term. The findings also suggest that avatar-based technology may support greater self-confidence, belief, and efficacy in weight loss ambitions. Overall, the trial design was found to be feasible and acceptable by participants and HCPs. Expansion of the inclusion criteria to include both primary care and local community weight management services may be beneficial. A full trial of the technology, taking into account participant feedback about the avatar technology design and accessibility, is also warranted.

## Appendix

Multimedia Appendix 1 CONSORT-eHEALTH checklist (V 1.6.1).

## Abbreviations

CONSORT-EHEALTH: Consolidated Standards of Reporting Trials of Electronic and Mobile Health Applications and Online Telehealth
HCP: health care professional
NHS: National Health Service
RCT: randomized controlled trial
VAS: Visual Analog Scale
VR: virtual reality
