# Supplementary material for: Using Personalized Avatars as an Adjunct to an Adult Weight Loss Management Program: Randomized Controlled Feasibility Study
Source: JMIR Form Res. 2022 Oct 5;6(10):e36275. doi: 10.2196/36275 (PMC9582922; doi:10.2196/36275)
Supplement: Multimedia Appendix 1 [file formative_v6i10e36275_app1.pdf]

CONSORT-EHEALTH (V 1.6.1) - Submission/Publication Form

The CONSORT-EHEALTH checklist is intended for authors of randomized trials evaluating web-based and Internet-based applications/interventions, including mobile interventions, electronic games (incl multiplayer games), social media, certain telehealth applications, and other interactive and/or networked electronic applications. Some of the items (e.g. all subitems under item 5 - description of the intervention) may also be applicable for other study designs.

The goal of the CONSORT EHEALTH checklist and guideline is to be
a) a guide for reporting for authors of RCTs,
b) to form a basis for appraisal of an ehealth trial (in terms of validity)

CONSORT-EHEALTH items/subitems are MANDATORY reporting items for studies published in the Journal of Medical Internet Research and other journals / scientific societies endorsing the checklist.

Items numbered 1., 2., 3., 4a., 4b etc are original CONSORT or CONSORT-NPT (non-pharmacologic treatment) items.
Items with Roman numerals (i., ii, iii, iv etc.) are CONSORT-EHEALTH extensions/clarifications.

As the CONSORT-EHEALTH checklist is still considered in a formative stage, we would ask that you also RATE ON A SCALE OF 1-5 how important/useful you feel each item is FOR THE PURPOSE OF THE CHECKLIST and reporting guideline (optional).

Mandatory reporting items are marked with a red *.
In the textboxes, either copy & paste the relevant sections from your manuscript into this form - please include any quotes from your manuscript in QUOTATION MARKS,
or answer directly by providing additional information not in the manuscript, or elaborating on why the item was not relevant for this study.

YOUR ANSWERS WILL BE PUBLISHED AS A SUPPLEMENTARY FILE TO YOUR PUBLICATION IN JMIR AND ARE CONSIDERED PART OF YOUR PUBLICATION (IF ACCEPTED).
Please fill in these questions diligently. Information will not be copyedited, so please use proper spelling and grammar, use correct capitalization, and avoid abbreviations.

DO NOT FORGET TO SAVE AS PDF _AND_ CLICK THE SUBMIT BUTTON SO YOUR ANSWERS ARE IN OUR DATABASE !!!

Citation Suggestion (if you append the pdf as Appendix we suggest to cite this paper in the caption):
Eysenbach G, CONSORT-EHEALTH Group
[illegible]

Your response is too large. Try shortening some answers.

[illegible]

URL: http://www.jmir.org/2011/4/e126/
doi: 10.2196/jmir.1923
PMID: 22209829

mariahorne19@gmail.com (not shared) Switch accounts

Draft not saved

*Required

Your name *

First Last

Maria Horne

Primary Affiliation (short), City, Country *

University of Toronto, Toronto, Canada

University of Leeds, Leeds, United Kingdom

Your e-mail address *

abc@gmail.com

M.Horne@Leeds.ac.uk

Title of your manuscript *

Provide the (draft) title of your manuscript.

Using personalised avatars as an adjunct to a weight loss management programme: a randomised controlled feasibility study

Your response is too large. Try shortening some answers.

Name of your App/Software/Intervention *

If there is a short and a long/alternate name, write the short name first and add the long name in brackets.

MotiVar

Evaluated Version (if any)

e.g. "V1", "Release 2017-03-01", "Version 2.0.27913"

JFR ms#36275.V1

Language(s) *

What language is the intervention/app in? If multiple languages are available, separate by comma (e.g. "English, French")

English

URL of your Intervention Website or App

e.g. a direct link to the mobile app on app in appstore (itunes, Google Play), or URL of the website. If the intervention is a DVD or hardware, you can also link to an Amazon page.

Your answer

URL of an image/screenshot (optional)

Your answer

Your response is too large. Try shortening some answers.

Accessibility *

Can an enduser access the intervention presently?

- ○ access is free and open
- ○ access only for special usergroups, not open
- ○ access is open to everyone, but requires payment/subscription/in-app purchases
- ○ app/intervention no longer accessible
- ◉ Other: the avatar was used in a feasibility study with a view to a full trial. Not

Primary Medical Indication/Disease/Condition *

e.g. "Stress", "Diabetes", or define the target group in brackets after the condition, e.g. "Autism (Parents of children with)", "Alzheimers (Informal Caregivers of)"

Obesity

Primary Outcomes measured in trial *

comma-separated list of primary outcomes reported in the trial

Weight loss using body weight (kg)

Secondary/other outcomes

Are there any other outcomes the intervention is expected to affect?

Uptake and continuation rates and changes in self-perceived quality of life and self-efficacy

Your response is too large. Try shortening some answers.

Recommended "Dose" *

What do the instructions for users say on how often the app should be used?

- ( ) Approximately Daily
- ( ) Approximately Weekly
- ( ) Approximately Monthly
- ( ) Approximately Yearly
- ( ) "as needed"
- (•) Other: Timed to coincide with the standard 12-week weight management prc

Approx. Percentage of Users (starters) still using the app as recommended after 3 months *

- ( ) unknown / not evaluated
- ( ) 0-10%
- ( ) 11-20%
- ( ) 21-30%
- ( ) 31-40%
- ( ) 41-50%
- ( ) 51-60%
- ( ) 61-70%
- ( ) 71%-80%
- ( ) 81-90%
- (•) 91-100%
- ( ) Other:

Your response is too large. Try shortening some answers.

Overall, was the app/intervention effective? *

- ○ yes: all primary outcomes were significantly better in intervention group vs control
- ○ partly: SOME primary outcomes were significantly better in intervention group vs control
- ○ no statistically significant difference between control and intervention
- ○ potentially harmful: control was significantly better than intervention in one or more outcomes
- ○ inconclusive: more research is needed
- ◉ Other: This was a feasibility study so assessing the effectiveness of the inte[illegible]

Article Preparation Status/Stage *

At which stage in your article preparation are you currently (at the time you fill in this form)

- ○ not submitted yet - in early draft status
- ○ not submitted yet - in late draft status, just before submission
- ○ submitted to a journal but not reviewed yet
- ○ submitted to a journal and after receiving initial reviewer comments
- ◉ submitted to a journal and accepted, but not published yet
- ○ published
- ○ Other:

Your response is too large. Try shortening some answers.

Journal *

If you already know where you will submit this paper (or if it is already submitted), please provide the journal name (if it is not JMIR, provide the journal name under "other")

- ( ) not submitted yet / unclear where I will submit this
- ( ) Journal of Medical Internet Research (JMIR)
- ( ) JMIR mHealth and UHealth
- ( ) JMIR Serious Games
- ( ) JMIR Mental Health
- ( ) JMIR Public Health
- (•) JMIR Formative Research
- ( ) Other JMIR sister journal
- ( ) Other:

Is this a full powered effectiveness trial or a pilot/feasibility trial? *

- (•) Pilot/feasibility
- ( ) Fully powered

Manuscript tracking number *

If this is a JMIR submission, please provide the manuscript tracking number under "other" (The ms tracking number can be found in the submission acknowledgement email, or when you login as author in JMIR. If the paper is already published in JMIR, then the ms tracking number is the four-digit number at the end of the DOI, to be found at the bottom of each published article in JMIR)

- ( ) no ms number (yet) / not (yet) submitted to / published in JMIR
- (•) Other: JFR ms#36275

Your response is too large. Try shortening some answers.

TITLE AND ABSTRACT

1a) TITLE: Identification as a randomized trial in the title

1a) Does your paper address CONSORT item 1a? *

I.e does the title contain the phrase "Randomized Controlled Trial"? (if not, explain the reason under "other")

- ○ yes
- ◉ Other: This study was a a randomised controlled feasibility study. This is clea

1a-i) Identify the mode of delivery in the title

Identify the mode of delivery. Preferably use “web-based” and/or “mobile” and/or “electronic game” in the title. Avoid ambiguous terms like “online”, “virtual”, “interactive”. Use “Internet-based” only if Intervention includes non-web-based Internet components (e.g. email), use “computer-based” or “electronic” only if offline products are used. Use “virtual” only in the context of “virtual reality” (3-D worlds). Use “online” only in the context of “online support groups”. Complement or substitute product names with broader terms for the class of products (such as “mobile” or “smart phone” instead of “iphone”), especially if the application runs on different platforms.

	1	2	3	4	5	
subitem not at all important	○	○	○	◉	○	essential

Clear selection

Your response is too large. Try shortening some answers.

Does your paper address subitem 1a-i? *

Copy and paste relevant sections from manuscript title (include quotes in quotation marks "like this" to indicate direct quotes from your manuscript), or elaborate on this item by providing additional information not in the ms, or briefly explain why the item is not applicable/relevant for your study

The title does not state that the personiised avatar is virtual but this is described iand discussed throughout the paper. However, an avatar is assumed to be virtual. Title stands currently as 'Using personalised avatars as an adjunct to a weight loss management programme: a randomised controlled feasibility study'.

1a-ii) Non-web-based components or important co-interventions in title

Mention non-web-based components or important co-interventions in title, if any (e.g., “with telephone support”).

	1	2	3	4	5	
subitem not at all important	○	○	○	●	○	essential

Clear selection

Does your paper address subitem 1a-ii?

Copy and paste relevant sections from manuscript title (include quotes in quotation marks "like this" to indicate direct quotes from your manuscript), or elaborate on this item by providing additional information not in the ms, or briefly explain why the item is not applicable/relevant for your study

Title states that this was as an adjucnt to "to a weight loss management programme"

Your response is too large. Try shortening some answers.

1a-iii) Primary condition or target group in the title

Mention primary condition or target group in the title, if any (e.g., “for children with Type I Diabetes”) Example: A Web-based and Mobile Intervention with Telephone Support for Children with Type I Diabetes: Randomized Controlled Trial

	1	2	3	4	5	
subitem not at all important	○	○	●	○	○	essential

Clear selection

Does your paper address subitem 1a-iii? *

Copy and paste relevant sections from manuscript title (include quotes in quotation marks "like this" to indicate direct quotes from your manuscript), or elaborate on this item by providing additional information not in the ms, or briefly explain why the item is not applicable/relevant for your study

The paper does not nquite address subitem 1a-iii. The title does outline that the avatar was an adjucnt to a weight manangement programme but does not state for overweight or obese individuals. This is outlined in the methods section under participants in the main manuscript. The title currently states: "Using personalised avatars as an adjunct to a weight loss management programme."

1b) ABSTRACT: Structured summary of trial design, methods, results, and conclusions

NPT extension: Description of experimental treatment, comparator, care providers, centers, and blinding status.

Your response is too large. Try shortening some answers.

1b-i) Key features/functionalities/components of the intervention and comparator in the METHODS section of the ABSTRACT

Mention key features/functionalities/components of the intervention and comparator in the abstract. If possible, also mention theories and principles used for designing the site. Keep in mind the needs of systematic reviewers and indexers by including important synonyms. (Note: Only report in the abstract what the main paper is reporting. If this information is missing from the main body of text, consider adding it)

	1	2	3	4	5	
subitem not at all important	○	○	○	◉	○	essential

Clear selection

Does your paper address subitem 1b-i? *

Copy and paste relevant sections from the manuscript abstract (include quotes in quotation marks "like this" to indicate direct quotes from your manuscript), or elaborate on this item by providing additional information not in the ms, or briefly explain why the item is not applicable/relevant for your study

The paper addresses subitem 1b-i. The key features in relation to the research approach has been mentioned as follows: "A feasibility randomised design was used to determine the case for future development and evaluation of avatar-based technology in a randomised controlled trial" and that the aim wsa to evaluate "...whether a personalised avatar, offered as an adjunct to an established weight loss programme" in the objective section.
The comparator is outlined in the results section of the abstract "Routine care plus avatar and routine care". It was not posible to mention theories and principles used for designing the avatar in the abtract re: word count limits but this aspect is discussed within the main manuscript in the methods section.

Your response is too large. Try shortening some answers.

1b-ii) Level of human involvement in the METHODS section of the ABSTRACT

Clarify the level of human involvement in the abstract, e.g., use phrases like “fully automated” vs. “therapist/nurse/care provider/physician-assisted” (mention number and expertise of providers involved, if any). (Note: Only report in the abstract what the main paper is reporting. If this information is missing from the main body of text, consider adding it)

	1	2	3	4	5	
subitem not at all important	○	○	◉	○	○	essential

Clear selection

Does your paper address subitem 1b-ii?

Copy and paste relevant sections from the manuscript abstract (include quotes in quotation marks "like this" to indicate direct quotes from your manuscript), or elaborate on this item by providing additional information not in the ms, or briefly explain why the item is not applicable/relevant for your study

The paper partially address's subitem 1b-ii. The level of involvement has not been specifically stated but the abstart does metionn that the intervention was an adjunct to a "GP referral, 12-week NHS weight improvement programme" in the methods section of the abstract.

Your response is too large. Try shortening some answers.

1b-iii) Open vs. closed, web-based (self-assessment) vs. face-to-face assessments in the METHODS section of the ABSTRACT

Mention how participants were recruited (online vs. offline), e.g., from an open access website or from a clinic or a closed online user group (closed usergroup trial), and clarify if this was a purely web-based trial, or there were face-to-face components (as part of the intervention or for assessment). Clearly say if outcomes were self-assessed through questionnaires (as common in web-based trials). Note: In traditional offline trials, an open trial (open-label trial) is a type of clinical trial in which both the researchers and participants know which treatment is being administered. To avoid confusion, use “blinded” or “unblinded” to indicated the level of blinding instead of “open”, as “open” in web-based trials usually refers to “open access” (i.e. participants can self-enrol). (Note: Only report in the abstract what the main paper is reporting. If this information is missing from the main body of text, consider adding it)

	1	2	3	4	5	
subitem not at all important	○	○	◉	○	○	essential

Clear selection

Does your paper address subitem 1b-iii?

Copy and paste relevant sections from the manuscript abstract (include quotes in quotation marks "like this" to indicate direct quotes from your manuscript), or elaborate on this item by providing additional information not in the ms, or briefly explain why the item is not applicable/relevant for your study

The methods of the abstract partially addresses subitem 1b-iii as follows: "Participants were recruited from GP referrals to a 12-week NHS weight improvement programme" which implies that this is undertaken offline via a clinic.
Participants in this feasibility study were not blinded.
Outcome measures are stated but not how specifcally collected in the abstract but discussed in the methods sectrion of the manuscript.

Your response is too large. Try shortening some answers.

1b-iv) RESULTS section in abstract must contain use data

Report number of participants enrolled/assessed in each group, the use/uptake of the intervention (e.g., attrition/adherence metrics, use over time, number of logins etc.), in addition to primary/secondary outcomes. (Note: Only report in the abstract what the main paper is reporting. If this information is missing from the main body of text, consider adding it)

	1	2	3	4	5	
subitem not at all important	○	○	○	○	◉	essential

Clear selection

Does your paper address subitem 1b-iv?

Copy and paste relevant sections from the manuscript abstract (include quotes in quotation marks "like this" to indicate direct quotes from your manuscript), or elaborate on this item by providing additional information not in the ms, or briefly explain why the item is not applicable/relevant for your study

The results section addresses subitem 1b-iv as follows: "10 males (7 routine care plus avatar; 3 routine care) and 33 females (23 intervention; 10 routine care) were recruited. Initial mean weight of participants was greater in the intervention than routine care (126.3 kg vs 122.9 kg); pattern of weight loss was similar across both arms of the study in period T0-T1 but accelerated in period T1-T2 for intervention participants, suggesting that access to the self-resembling avatar may promote greater engagement with weight loss initiatives in the short to medium term. Mean change in weight of participants from T0 to T2 was 4.5kg (95% CI: 2.7-6.3) in routine care arm and 5.3kg (95% CI: 3.9-6.8) in the intervention arm. Quality-of-life and self-efficacy measures demonstrated greater improvement in the intervention arm at both T1 (105.5 routine care; 99.7 intervention arm) and T2 (100.1 routine care; 81.2 intervention arm). 13 participants (11 Female, 2 Male) and two healthcare professionals were interviewed about their experience of using the avatar programme".

Your response is too large. Try shortening some answers.

1b-v) CONCLUSIONS/DISCUSSION in abstract for negative trials

Conclusions/Discussions in abstract for negative trials: Discuss the primary outcome - if the trial is negative (primary outcome not changed), and the intervention was not used, discuss whether negative results are attributable to lack of uptake and discuss reasons. (Note: Only report in the abstract what the main paper is reporting. If this information is missing from the main body of text, consider adding it)

	1	2	3	4	5	
subitem not at all important	(●)	○	○	○	○	essential

Clear selection

Does your paper address subitem 1b-v?

Copy and paste relevant sections from the manuscript abstract (include quotes in quotation marks "like this" to indicate direct quotes from your manuscript), or elaborate on this item by providing additional information not in the ms, or briefly explain why the item is not applicable/relevant for your study

This feasibikity was not a negative trial. The conclusion only reeports what is in the main paper.

INTRODUCTION

2a) In INTRODUCTION: Scientific background and explanation of rationale

Your response is too large. Try shortening some answers.

2a-i) Problem and the type of system/solution

Describe the problem and the type of system/solution that is object of the study: intended as stand-alone intervention vs. incorporated in broader health care program? Intended for a particular patient population? Goals of the intervention, e.g., being more cost-effective to other interventions, replace or complement other solutions? (Note: Details about the intervention are provided in “Methods” under 5)

	1	2	3	4	5	
subitem not at all important	○	○	○	○	◉	essential

Clear selection

Does your paper address subitem 2a-i? *

Copy and paste relevant sections from the manuscript (include quotes in quotation marks "like this" to indicate direct quotes from your manuscript), or elaborate on this item by providing additional information not in the ms, or briefly explain why the item is not applicable/relevant for your study

The paper addrresses subitem 2a-i. The problem and its impact has been described in the first paragraph of the introduction. Technology based interventions for weight loss have been discussed and how this intervention, by "Transforming the appearance of self through an avatar is a particularly powerful motivational application" in weight management but that "there has been limited translation of this technology into healthcare service delivery".

2a-ii) Scientific background, rationale: What is known about the (type of) system

Scientific background, rationale: What is known about the (type of) system that is the object of the study (be sure to discuss the use of similar systems for other conditions/diagnoses, if appropiate), motivation for the study, i.e. what are the reasons for and what is the context for this specific study, from which stakeholder viewpoint is the study performed, potential impact of findings [2]. Briefly justify the choice of the comparator.

	1	2	3	4	5	
subitem not at all important	○	○	○	○	◉	essential

Clear selection

Your response is too large. Try shortening some answers.

Does your paper address subitem 2a-ii? *

Copy and paste relevant sections from the manuscript (include quotes in quotation marks "like this" to indicate direct quotes from your manuscript), or elaborate on this item by providing additional information not in the ms, or briefly explain why the item is not applicable/relevant for your study

The paper addresses subitem 2a-ii as it does discuss the scientific background and rationale as follows: "Technology based interventions for weight loss have shown promise in the short-term [15,16], but the potential of computer engineering has not been fully realised when designing weight loss interventions [17]. It has been argued that new multicomponent (integrated) electronic platforms integrating education with individually tailored weight loss programmes, including the promotion of autonomous motivation, self-efficacy and self-regulation, as well as positive body image, may present a way forward for wide scale weight loss solutions and obesity management [18,19,20]. One area that has significant potential to be exploited as a catalyst for weight loss behaviour modification within healthcare is virtual reality (VR) and the creation of personal avatars [15]. Virtual reality (VR) enables people to experience an alternate visual reality, often through an avatar (computerised representation of self). Avatar technology is well established within the computer gaming industry and third person perspective of self within a VR setting has been shown to promote emotional engagement [21,22]".
The theorectical background has beed discussed: "Based on Bandura's social cognitive theory [23] (behaviour learning through observation of models) and Bem's self-perception theory [24] (inference of own attitudes by observing self from third party perspective), research has demonstrated a link between virtual representations of self and real-world behaviour and attitudes, as a consequence of individuals identifying with their avatar [25, 26]; particularly where the avatar is self-resembling [27] and experience of presence within the computer mediated environment [28].
The motivation for the feasbility study has also been disucssed as follows: "The potential of virtual reality as an intervention has been explored across a spectrum of health and well-being conditions [25, 26, 29,30] and it has been recommended as a potential behaviour modification tool for tackling obesity [31, 32]. Studies have shown that within the experimental environment, an individual's behavior conforms to their digital self-representation (Proteus effect) [33,34,35]. Importantly, observing a self-resembling avatar modelling an activity (e.g. exercise) within a VR environment, as opposed to a generic avatar, differentially positively influences real world behavior, increasing actual engagement with the activity [29, 36]. This connectedness between actual and digital self has been further explored using ageing algorithms to present individuals with personalized virtual futures influenced by choices made today [37, 38] and results suggest that observation of future self within a virtual environment may vicariously reinforce today's desirable behaviors and attitudes".
The choice of the comparator has not been discussed in this section.

Your response is too large. Try shortening some answers.

Does your paper address CONSORT subitem 2b? *

Copy and paste relevant sections from the manuscript (include quotes in quotation marks "like this" to indicate direct quotes from your manuscript), or elaborate on this item by providing additional information not in the ms, or briefly explain why the item is not applicable/relevant for your study

The feasibility paper addresses CONSORT subitem 2b as follows: "We proposed to develop a self-resembling avatar generation programme to allow individuals to explore potential health futures related to obesity, including observing their self-avatar gain and lose weight and with information on the associated health risks at BMI boundaries. This feasibility study tested the avatar design capabilities, clinical application and the underlying premise that this technology, provided as an adjunct to an existing weight loss programme, would positively influence participant motivation".

METHODS

3a) Description of trial design (such as parallel, factorial) including allocation ratio

Does your paper address CONSORT subitem 3a? *

Copy and paste relevant sections from the manuscript (include quotes in quotation marks "like this" to indicate direct quotes from your manuscript), or elaborate on this item by providing additional information not in the ms, or briefly explain why the item is not applicable/relevant for your study

The fesability study paper addresses CONSORT subitem 3a as follows in the methods section: "This was a two phase, sequential, mixed methods feasibility study, incorporating a feasibility randomised controlled trial (RCT) with a subsequent qualitative component, to determine the case for, and the parameters of, a future RCT".

3b) Important changes to methods after trial commencement (such as eligibility criteria), with reasons

Your response is too large. Try shortening some answers.

Does your paper address CONSORT subitem 3b? *

Copy and paste relevant sections from the manuscript (include quotes in quotation marks "like this" to indicate direct quotes from your manuscript), or elaborate on this item by providing additional information not in the ms, or briefly explain why the item is not applicable/relevant for your study

The paper addresses CONSORT subitem 3b. As a result of the COVID pandemic changes in service delivery from face-to-face to telephone appointments were made. This has been stated in the results section of the paper as follows: "....restrictions and changes in service delivery from face-to-face to telephone appointments as a result of the COVID pandemic influenced the final stages of data collection. As a result, T3 (6 month) data were not collected for the majority of participants and analysis was restricted to T0-T2".

3b-i) Bug fixes, Downtimes, Content Changes

Bug fixes, Downtimes, Content Changes: ehealth systems are often dynamic systems. A description of changes to methods therefore also includes important changes made on the intervention or comparator during the trial (e.g., major bug fixes or changes in the functionality or content) (5-iii) and other "unexpected events" that may have influenced study design such as staff changes, system failures/downtimes, etc. [2].

	1	2	3	4	5	
subitem not at all important	(•)	( )	( )	( )	( )	essential

Clear selection

Does your paper address subitem 3b-i?

Copy and paste relevant sections from the manuscript (include quotes in quotation marks "like this" to indicate direct quotes from your manuscript), or elaborate on this item by providing additional information not in the ms, or briefly explain why the item is not applicable/relevant for your study

There were no Bug fixes, Downtimes, Content Changes with this intervention during the fesability study.

4a) Eligibility criteria for participants

Your response is too large. Try shortening some answers.

Does your paper address CONSORT subitem 4a? *

Copy and paste relevant sections from the manuscript (include quotes in quotation marks "like this" to indicate direct quotes from your manuscript), or elaborate on this item by providing additional information not in the ms, or briefly explain why the item is not applicable/relevant for your study

The paper addresses CONSORT subitem 4a as follows in relation to the eligibility criteria for participants: "Participants were patients with obesity or were overweight from within West Yorkshire, England, referred to the Mid Yorkshire Hospitals NHS Trust weight improvement service (March 2019-December 2019). Inclusion criteria were: (i) adults aged 18-65 years; (ii) BMI >30; (iii) referred from GP; (iv) no known co-morbidities or medical treatment that may influence dietary intake or weight loss achievement (e.g. type I diabetes; current medication with known weight gain side effect).
Exclusion criteria were: (i) pregnant women; (ii) children; (iii) older persons (>65 years); (iv) BMI >45; (v) persons considered psychologically vulnerable".

4a-i) Computer / Internet literacy

Computer / Internet literacy is often an implicit “de facto” eligibility criterion - this should be explicitly clarified.

	1	2	3	4	5	
subitem not at all important	◉	○	○	○	○	essential

Clear selection

Does your paper address subitem 4a-i?

Copy and paste relevant sections from the manuscript (include quotes in quotation marks "like this" to indicate direct quotes from your manuscript), or elaborate on this item by providing additional information not in the ms, or briefly explain why the item is not applicable/relevant for your study

The paper does not address subitem 4a-i around computer literacy but states that: "..changes in avatar appearance were presented and discussed with the participants at each researcher appointment and participants were encouraged to use the avatar to visualise progress" in the intervention section.

Your response is too large. Try shortening some answers.

4a-ii) Open vs. closed, web-based vs. face-to-face assessments:

Open vs. closed, web-based vs. face-to-face assessments: Mention how participants were recruited (online vs. offline), e.g., from an open access website or from a clinic, and clarify if this was a purely web-based trial, or there were face-to-face components (as part of the intervention or for assessment), i.e., to what degree got the study team to know the participant. In online-only trials, clarify if participants were quasi-anonymous and whether having multiple identities was possible or whether technical or logistical measures (e.g., cookies, email confirmation, phone calls) were used to detect/prevent these.

	1	2	3	4	5	
subitem not at all important	○	○	◉	○	○	essential

Clear selection

Your response is too large. Try shortening some answers.

Does your paper address subitem 4a-ii? *

Copy and paste relevant sections from the manuscript (include quotes in quotation marks "like this" to indicate direct quotes from your manuscript), or elaborate on this item by providing additional information not in the ms, or briefly explain why the item is not applicable/relevant for your study

The paper partially addresses subitem 4a-ii. This was not a web-based feasibility study buyt there were face-to-face components (as part of the intervention and assessment). This is outlined. This is dicussed in the intervention section as follows: "The weight management routine care programme operated over 12-weeks. Patients who were allocated to receive the intervention (routine care plus avatar) attended the weight improvement service for their initial appointment (T0). Additional time was allocated to this appointment to meet the researcher and for baseline anthropomorphic measures and digital photographs to be acquired. These parameters were inputted into the avatar creation programme while participants completed three questionnaires: (i) Impact of Weight on Quality of Life-lite (IWQOL-lite) [39]; (ii) weight efficacy lifestyle-short form questionnaire (WEL-SF) [40]; and EQ-5D-5L [41] to monitor changes in wellbeing during the study period and identify the best tool to be adopted in a future RCT if warranted. Once the avatar was generated (less than 5 minutes post data input), the participant was introduced to their avatar and programme operation and given a unique username and password to allow them to access their avatar online via computer or tablet. Participants were encouraged to explore how increasing/decreasing weight impacts on avatar appearance, consider 5% and 10% weight loss goals promoted by the weight management routine care programme alongside the BMI boundary health risks as their avatar increased and decreased in weight.

Follow-up appointments with the researcher coincided with weight management service routine care appointments at 1-month (T1) (immediate effect) and 3-month (T2) (programme completion short term effect). A further appointment to meet with the researcher was planned for 6 months (T3) (3-month post-programme completion - moderate term progress). At each of these appointments the researcher collected completed surveys and inputted any change in weight into the avatar programme. The changes in avatar appearance were presented and discussed with the participants at each researcher appointment and participants were encouraged to use the avatar to visualise progress" and under routine care as follows: "Routine care was the standard 12-week weight management programme without provision of an avatar. Participants allocated to receive routine care were given the same number of appointments with the weight improvement service as those allocated to the intervention group and met the researcher to complete questionnaires and acquire body measurements, photographs and weight change data at the same points as those participating in the intervention arm. However, while routine care participants underwent the same process of meeting the researcher and replicating activity of the intervention, visualisation of, and access to, their avatar was withheld until the final weight management service appointment (12-week)".

Your response is too large. Try shortening some answers.

4a-iii) Information giving during recruitment

Information given during recruitment. Specify how participants were briefed for recruitment and in the informed consent procedures (e.g., publish the informed consent documentation as appendix, see also item X26), as this information may have an effect on user self-selection, user expectation and may also bias results.

	1	2	3	4	5	
subitem not at all important	○	○	○	●	○	essential

Clear selection

Does your paper address subitem 4a-iii?

Copy and paste relevant sections from the manuscript (include quotes in quotation marks "like this" to indicate direct quotes from your manuscript), or elaborate on this item by providing additional information not in the ms, or briefly explain why the item is not applicable/relevant for your study

The feasibility study paper addresses subitem 4a-iii. How participants were briefed for recruitment and in the informed consent procedures have been discussed under recruitment as follows: "Potential participants were identified by the weight management service clinical team (Mid Yorkshire Hospitals NHS Trust) following initial assessment of suitability to participate in a weight management programme and study participation screening. Potential participants were given an information pack, including invitation letter, information sheet, researcher permission to contact form and postage paid envelope to read, complete and return after the appointment. The forms were returned to the weight management service and a secure database of potential participants created by the NHS research administrator assigned to this project. The researcher confirmed with the administrator on a weekly basis those patients who had returned the permission to contact forms and telephoned those patients interested in participating to describe the study further and confirm willingness to participate" and in the ethical review section as follows: "All participants were screened for suitability by a registered medical clinician specialising in obesity including assessment of psychological vulnerability. All participants provided written informed consent and a process of medical referral and reporting of any potential harm as a result of the study participation established".

4b) Settings and locations where the data were collected

Your response is too large. Try shortening some answers.

Does your paper address CONSORT subitem 4b? *

Copy and paste relevant sections from the manuscript (include quotes in quotation marks "like this" to indicate direct quotes from your manuscript), or elaborate on this item by providing additional information not in the ms, or briefly explain why the item is not applicable/relevant for your study

The paper addresses CONSORT subitem 4b as follows under the recruitment section: "Potential participants were identified by the weight management service clinical team (Mid Yorkshire Hospitals NHS Trust)" and, re the intervention development, in the intervention section: "Service user volunteers were recruited from the University of Bradford service user group and HCP participants were recruited from Mid Yorkshire Hospitals NHS Trust weight management service".

4b-i) Report if outcomes were (self-)assessed through online questionnaires

Clearly report if outcomes were (self-)assessed through online questionnaires (as common in web-based trials) or otherwise.

	1	2	3	4	5	
subitem not at all important	◉	○	○	○	○	essential

Clear selection

Does your paper address subitem 4b-i? *

Copy and paste relevant sections from the manuscript (include quotes in quotation marks "like this" to indicate direct quotes from your manuscript), or elaborate on this item by providing additional information not in the ms, or briefly explain why the item is not applicable/relevant for your study

The paper does not address subitem 4b-i as outcomes were not (self-)assessed through online questionnaires.

Your response is too large. Try shortening some answers.

4b-ii) Report how institutional affiliations are displayed

Report how institutional affiliations are displayed to potential participants [on ehealth media], as affiliations with prestigious hospitals or universities may affect volunteer rates, use, and reactions with regards to an intervention.(Not a required item – describe only if this may bias results)

	1	2	3	4	5	
subitem not at all important	◉	○	○	○	○	essential

Clear selection

Does your paper address subitem 4b-ii?

Copy and paste relevant sections from the manuscript (include quotes in quotation marks "like this" to indicate direct quotes from your manuscript), or elaborate on this item by providing additional information not in the ms, or briefly explain why the item is not applicable/relevant for your study

The paper does not address subitem 4b-ii. Institutional affiliations were displayed on patient information and consent sheets. This was felt not to bias the results of the feasibility study.

5) The interventions for each group with sufficient details to allow replication, including how and when they were actually administered

5-i) Mention names, credential, affiliations of the developers, sponsors, and owners

Mention names, credential, affiliations of the developers, sponsors, and owners [6] (if authors/evaluators are owners or developer of the software, this needs to be declared in a "Conflict of interest" section or mentioned elsewhere in the manuscript).

	1	2	3	4	5	
subitem not at all important	○	◉	○	○	○	essential

Clear selection

Your response is too large. Try shortening some answers.

Does your paper address subitem 5-i?

Copy and paste relevant sections from the manuscript (include quotes in quotation marks "like this" to indicate direct quotes from your manuscript), or elaborate on this item by providing additional information not in the ms, or briefly explain why the item is not applicable/relevant for your study

The paper addresses subitem 5-i as follows in the intervention section: "The avatar virtual reality programme (creation and display) was developed through an iterative design process with service users and healthcare professionals (HCP). Service user volunteers were recruited from the University of Bradford service user group and HCP participants were recruited from Mid Yorkshire Hospitals NHS Trust weight management service. Feedback at each stage of development ensured that the final avatar programme design was optimised prior to commencing the feasibility study". There were no conflcits of interest.

5-ii) Describe the history/development process

Describe the history/development process of the application and previous formative evaluations (e.g., focus groups, usability testing), as these will have an impact on adoption/use rates and help with interpreting results.

	1	2	3	4	5	
subitem not at all important	○	○	○	○	◉	essential

Clear selection

Does your paper address subitem 5-ii?

Copy and paste relevant sections from the manuscript (include quotes in quotation marks "like this" to indicate direct quotes from your manuscript), or elaborate on this item by providing additional information not in the ms, or briefly explain why the item is not applicable/relevant for your study

The feasibility paper addresses subitem 5-ii as follows in the intervention section as follows: "The avatar virtual reality programme (creation and display) was developed through an iterative design process with service users and healthcare professionals (HCP). Service user volunteers were recruited from the University of Bradford service user group and HCP participants were recruited from Mid Yorkshire Hospitals NHS Trust weight management service. Feedback at each stage of development ensured that the final avatar programme design was optimised prior to commencing the feasibility study".

Your response is too large. Try shortening some answers.

5-iii) Revisions and updating

Revisions and updating. Clearly mention the date and/or version number of the application/intervention (and comparator, if applicable) evaluated, or describe whether the intervention underwent major changes during the evaluation process, or whether the development and/or content was “frozen” during the trial. Describe dynamic components such as news feeds or changing content which may have an impact on the replicability of the intervention (for unexpected events see item 3b).

	1	2	3	4	5	
subitem not at all important	○	○	◉	○	○	essential

Clear selection

Does your paper address subitem 5-iii?

Copy and paste relevant sections from the manuscript (include quotes in quotation marks "like this" to indicate direct quotes from your manuscript), or elaborate on this item by providing additional information not in the ms, or briefly explain why the item is not applicable/relevant for your study

There were no revisions of the final avatar implemeted in this feasibility study. The version used was being evaluated and from this revions would potential be made.

5-iv) Quality assurance methods

Provide information on quality assurance methods to ensure accuracy and quality of information provided [1], if applicable.

	1	2	3	4	5	
subitem not at all important	○	○	○	◉	○	essential

Clear selection

Your response is too large. Try shortening some answers.

Does your paper address subitem 5-iv?

Copy and paste relevant sections from the manuscript (include quotes in quotation marks "like this" to indicate direct quotes from your manuscript), or elaborate on this item by providing additional information not in the ms, or briefly explain why the item is not applicable/relevant for your study

The paper addresses subitem 5-iv in the data analysis section of the feasibility paper as follows: "Data analysis focused on describing the key feasibility outcomes using descriptive statistical tests including calculation of means and frequencies. Mean (standard deviation, 95%confidence interval) and median (inter-quartile range) values were reported by study arm (routine care and routine care plus avatar) for the primary outcomes of weight (kg) and body mass index and secondary effectiveness outcome data collected through the questionnaires. A generalised linear repeated measures) mixed effects model (GLMM) for weight (kg) was used to account for the discrete timing of the follow-up assessments and adjusting for baseline measure (T0). The aim of the GLMM was to obtain an estimate of variability (residual standard deviation) for weight change, and to estimate mean change in weight (95% confidence interval) from baseline (TO) to T1 and T2, that would then be used to inform the sample size calculation for a future definitive trial if warranted.

Qualitative data were analysed using framework approach [44]. The first author coded all transcripts. Codes were then discussed with the research team who together developed the indexing scheme that was used by the first author to chart data. These charts were shared with others in the team to explore and interpret the data together and to decide on the final themes. Consensus on themes and subthemes was reached through discussion".

5-v) Ensure replicability by publishing the source code, and/or providing screenshots/screen-capture video, and/or providing flowcharts of the algorithms used

Ensure replicability by publishing the source code, and/or providing screenshots/screen-capture video, and/or providing flowcharts of the algorithms used. Replicability (i.e., other researchers should in principle be able to replicate the study) is a hallmark of scientific reporting.

	1	2	3	4	5	
subitem not at all important	○	○	○	○	◉	essential

Clear selection

Your response is too large. Try shortening some answers.

Does your paper address subitem 5-v?

Copy and paste relevant sections from the manuscript (include quotes in quotation marks "like this" to indicate direct quotes from your manuscript), or elaborate on this item by providing additional information not in the ms, or briefly explain why the item is not applicable/relevant for your study

The paper addresses subitem 5-v throughout the methods section. Source code, and/or screenshots/screen-capture video are not applicable.

5-vi) Digital preservation

Digital preservation: Provide the URL of the application, but as the intervention is likely to change or disappear over the course of the years; also make sure the intervention is archived (Internet Archive, webcitation.org, and/or publishing the source code or screenshots/videos alongside the article). As pages behind login screens cannot be archived, consider creating demo pages which are accessible without login.

	1	2	3	4	5	
subitem not at all important	◉	○	○	○	○	essential

Clear selection

Does your paper address subitem 5-vi?

Copy and paste relevant sections from the manuscript (include quotes in quotation marks "like this" to indicate direct quotes from your manuscript), or elaborate on this item by providing additional information not in the ms, or briefly explain why the item is not applicable/relevant for your study

This feasibility study evaulated/tested the avatar design capabilities, clinical application and the underlying premise that this technology, provided as an adjunct to an existing weight loss programme, would positively influence participant motivation and is subject to change based on the findings/results.

Your response is too large. Try shortening some answers.

5-vii) Access

Access: Describe how participants accessed the application, in what setting/context, if they had to pay (or were paid) or not, whether they had to be a member of specific group. If known, describe how participants obtained “access to the platform and Internet” [1]. To ensure access for editors/reviewers/readers, consider to provide a “backdoor” login account or demo mode for reviewers/readers to explore the application (also important for archiving purposes, see vi).

	1	2	3	4	5	
subitem not at all important	○	○	○	○	◉	essential

Clear selection

Does your paper address subitem 5-vii? *

Copy and paste relevant sections from the manuscript (include quotes in quotation marks "like this" to indicate direct quotes from your manuscript), or elaborate on this item by providing additional information not in the ms, or briefly explain why the item is not applicable/relevant for your study

The paper addresses subitem 5-vii under the intervention section as follows: "Once the avatar was generated (less than 5 minutes post data input), the participant was introduced to their avatar and programme operation and given a unique username and password to allow them to access their avatar online via computer or tablet. Participants were encouraged to explore how increasing/decreasing weight impacts on avatar appearance, consider 5% and 10% weight loss goals promoted by the weight management routine care programme alongside the BMI boundary health risks as their avatar increased and decreased in weight.

Follow-up appointments with the researcher coincided with weight management service routine care appointments at 1-month (T1) (immediate effect) and 3-month (T2) (programme completion short term effect). A further appointment to meet with the researcher was planned for 6 months (T3) (3-month post-programme completion - moderate term progress). At each of these appointments the researcher collected completed surveys and inputted any change in weight into the avatar programme. The changes in avatar appearance were presented and discussed with the participants at each researcher appointment and participants were encouraged to use the avatar to visualise progress".

Your response is too large. Try shortening some answers.

5-viii) Mode of delivery, features/functionalities/components of the intervention and comparator, and the theoretical framework

Describe mode of delivery, features/functionalities/components of the intervention and comparator, and the theoretical framework [6] used to design them (instructional strategy [1], behaviour change techniques, persuasive features, etc., see e.g., [7, 8] for terminology). This includes an in-depth description of the content (including where it is coming from and who developed it) [1]," whether [and how] it is tailored to individual circumstances and allows users to track their progress and receive feedback" [6]. This also includes a description of communication delivery channels and – if computer-mediated communication is a component – whether communication was synchronous or asynchronous [6]. It also includes information on presentation strategies [1], including page design principles, average amount of text on pages, presence of hyperlinks to other resources, etc. [1].

	1	2	3	4	5	
subitem not at all important	○	○	○	○	◉	essential

Clear selection

Does your paper address subitem 5-viii? *

Copy and paste relevant sections from the manuscript (include quotes in quotation marks "like this" to indicate direct quotes from your manuscript), or elaborate on this item by providing additional information not in the ms, or briefly explain why the item is not applicable/relevant for your study

The paper addresses subitem 5-vii. The mode of delivery, features/functionalities/components of the intervention and comparator are addrewssed i b the methods section. The underpinning theoretical framework and behaviour change techniques are discussed in the introduction - Secondary outcome measures were uptake and continuation rates and changes in self-perceived quality of life and self-efficacy. These have been discussed in the outcomes section.

Your response is too large. Try shortening some answers.

5-ix) Describe use parameters

Describe use parameters (e.g., intended “doses” and optimal timing for use). Clarify what instructions or recommendations were given to the user, e.g., regarding timing, frequency, heaviness of use, if any, or was the intervention used ad libitum.

	1	2	3	4	5	
subitem not at all important	○	○	○	○	◉	essential

Clear selection

Your response is too large. Try shortening some answers.

Does your paper address subitem 5-ix?

Copy and paste relevant sections from the manuscript (include quotes in quotation marks "like this" to indicate direct quotes from your manuscript), or elaborate on this item by providing additional information not in the ms, or briefly explain why the item is not applicable/relevant for your study

The feasibility paper addresses subitem 5-ix. This is outlined in the methods section as follows: "The weight management routine care programme operated over 12-weeks. Patients who were allocated to receive the intervention (routine care plus avatar) attended the weight improvement service for their initial appointment (T0). Additional time was allocated to this appointment to meet the researcher and for baseline anthropomorphic measures and digital photographs to be acquired. These parameters were inputted into the avatar creation programme while participants completed three questionnaires: (i) Impact of Weight on Quality of Life-lite (IWQOL-lite) [39]; (ii) weight efficacy lifestyle-short form questionnaire (WEL-SF) [40]; and EQ-5D-5L [41] to monitor changes in wellbeing during the study period and identify the best tool to be adopted in a future RCT if warranted. Once the avatar was generated (less than 5 minutes post data input), the participant was introduced to their avatar and programme operation and given a unique username and password to allow them to access their avatar online via computer or tablet. Participants were encouraged to explore how increasing/decreasing weight impacts on avatar appearance, consider 5% and 10% weight loss goals promoted by the weight management routine care programme alongside the BMI boundary health risks as their avatar increased and decreased in weight.

Follow-up appointments with the researcher coincided with weight management service routine care appointments at 1-month (T1) (immediate effect) and 3-month (T2) (programme completion short term effect). A further appointment to meet with the researcher was planned for 6 months (T3) (3-month post-programme completion - moderate term progress). At each of these appointments the researcher collected completed surveys and inputted any change in weight into the avatar programme. The changes in avatar appearance were presented and discussed with the participants at each researcher appointment and participants were encouraged to use the avatar to visualise progress.

Routine Care
Routine care was the standard 12-week weight management programme without provision of an avatar. Participants allocated to receive routine care were given the same number of appointments with the weight improvement service as those allocated to the intervention group and met the researcher to complete questionnaires and acquire body measurements, photographs and weight change data at the same points as those participating in the intervention arm. However, while routine care participants underwent the same process of meeting the researcher and replicating activity of the intervention, visualisation of, and access to, their avatar was withheld until the final weight management service appointment (12-week)".

Your response is too large. Try shortening some answers.

5-x) Clarify the level of human involvement

Clarify the level of human involvement (care providers or health professionals, also technical assistance) in the e-intervention or as co-intervention (detail number and expertise of professionals involved, if any, as well as “type of assistance offered, the timing and frequency of the support, how it is initiated, and the medium by which the assistance is delivered”. It may be necessary to distinguish between the level of human involvement required for the trial, and the level of human involvement required for a routine application outside of a RCT setting (discuss under item 21 – generalizability).

	1	2	3	4	5	
subitem not at all important	○	○	○	◉	○	essential

Clear selection

Your response is too large. Try shortening some answers.

Does your paper address subitem 5-x?

Copy and paste relevant sections from the manuscript (include quotes in quotation marks "like this" to indicate direct quotes from your manuscript), or elaborate on this item by providing additional information not in the ms, or briefly explain why the item is not applicable/relevant for your study

The paper addresses subitem 5-x in that it is outlined that this was based on a GP referral weight management programme. In the intervention section it is stated that: "The weight management routine care programme operated over 12-weeks. Patients who were allocated to receive the intervention (routine care plus avatar) attended the weight improvement service for their initial appointment (T0). Additional time was allocated to this appointment to meet the researcher and for baseline anthropomorphic measures and digital photographs to be acquired. These parameters were inputted into the avatar creation programme while participants completed three questionnaires: (i) Impact of Weight on Quality of Life-lite (IWQOL-lite) [39]; (ii) weight efficacy lifestyle-short form questionnaire (WEL-SF) [40]; and EQ-5D-5L [41] to monitor changes in wellbeing during the study period and identify the best tool to be adopted in a future RCT if warranted. Once the avatar was generated (less than 5 minutes post data input), the participant was introduced to their avatar and programme operation and given a unique username and password to allow them to access their avatar online via computer or tablet. Participants were encouraged to explore how increasing/decreasing weight impacts on avatar appearance, consider 5% and 10% weight loss goals promoted by the weight management routine care programme alongside the BMI boundary health risks as their avatar increased and decreased in weight.

Follow-up appointments with the researcher coincided with weight management service routine care appointments at 1-month (T1) (immediate effect) and 3-month (T2) (programme completion short term effect). A further appointment to meet with the researcher was planned for 6 months (T3) (3-month post-programme completion - moderate term progress). At each of these appointments the researcher collected completed surveys and inputted any change in weight into the avatar programme. The changes in avatar appearance were presented and discussed with the participants at each researcher appointment and participants were encouraged to use the avatar to visualise progress.

Routine Care
Routine care was the standard 12-week weight management programme without provision of an avatar. Participants allocated to receive routine care were given the same number of appointments with the weight improvement service as those allocated to the intervention group and met the researcher to complete questionnaires and acquire body measurements, photographs and weight change data at the same points as those participating in the intervention arm. However, while routine care participants underwent the same process of meeting the researcher and replicating activity of the intervention, visualisation of, and access to, their avatar was withheld until the final weight management service appointment (12-week)".

Your response is too large. Try shortening some answers.

5-xi) Report any prompts/reminders used

Report any prompts/reminders used: Clarify if there were prompts (letters, emails, phone calls, SMS) to use the application, what triggered them, frequency etc. It may be necessary to distinguish between the level of prompts/reminders required for the trial, and the level of prompts/reminders for a routine application outside of a RCT setting (discuss under item 21 – generalizability).

	1	2	3	4	5	
subitem not at all important	◉	○	○	○	○	essential

Clear selection

Does your paper address subitem 5-xi? *

Copy and paste relevant sections from the manuscript (include quotes in quotation marks "like this" to indicate direct quotes from your manuscript), or elaborate on this item by providing additional information not in the ms, or briefly explain why the item is not applicable/relevant for your study

The paper does not address subitem 5-x as no further prompts (letters, emails, phone calls, SMS) to use the avatar were provisded.

5-xii) Describe any co-interventions (incl. training/support)

Describe any co-interventions (incl. training/support): Clearly state any interventions that are provided in addition to the targeted eHealth intervention, as ehealth intervention may not be designed as stand-alone intervention. This includes training sessions and support [1]. It may be necessary to distinguish between the level of training required for the trial, and the level of training for a routine application outside of a RCT setting (discuss under item 21 – generalizability.

	1	2	3	4	5	
subitem not at all important	◉	○	○	○	○	essential

Clear selection

Your response is too large. Try shortening some answers.

Does your paper address subitem 5-xii? *

Copy and paste relevant sections from the manuscript (include quotes in quotation marks "like this" to indicate direct quotes from your manuscript), or elaborate on this item by providing additional information not in the ms, or briefly explain why the item is not applicable/relevant for your study

The paper does not address subitem 5-xii as no co-interventions (incl. training/support) provided.

6a) Completely defined pre-specified primary and secondary outcome measures, including how and when they were assessed

Does your paper address CONSORT subitem 6a? *

Copy and paste relevant sections from the manuscript (include quotes in quotation marks "like this" to indicate direct quotes from your manuscript), or elaborate on this item by providing additional information not in the ms, or briefly explain why the item is not applicable/relevant for your study

The paper addresses CONSORT subitem 6a by stating the primary and secondary outcome measures in the Outcome measures section as follows:
"The primary outcome measure was weight loss using body weight (kg) and calculated BMI (kg/m2). Assessment of weight loss was planned for baseline, three and six months. Secondary outcome measures were uptake and continuation rates and changes in self-perceived quality of life and self-efficacy".
How and when the primary and secondary outcome measures were assessed are outlined in the intervention section as follows: "The weight management routine care programme operated over 12-weeks. Patients who were allocated to receive the intervention (routine care plus avatar) attended the weight improvement service for their initial appointment (T0). Additional time was allocated to this appointment to meet the researcher and for baseline anthropomorphic measures and digital photographs to be acquired. These parameters were inputted into the avatar creation programme while participants completed three questionnaires: (i) Impact of Weight on Quality of Life-lite (IWQOL-lite) [39]; (ii) weight efficacy lifestyle-short form questionnaire (WEL-SF) [40]; and EQ-5D-5L [41] to monitor changes in wellbeing during the study period and identify the best tool to be adopted in a future RCT if warranted".

Your response is too large. Try shortening some answers.

6a-i) Online questionnaires: describe if they were validated for online use and apply CHERRIES items to describe how the questionnaires were designed/deployed

If outcomes were obtained through online questionnaires, describe if they were validated for online use and apply CHERRIES items to describe how the questionnaires were designed/deployed [9].

	1	2	3	4	5	
subitem not at all important	◉	○	○	○	○	essential

Clear selection

Does your paper address subitem 6a-i?

Copy and paste relevant sections from manuscript text

Online questionnaires were not utlised. Validated paper based questionnaires were used and outlined in intervention section as follows: "Additional time was allocated to this appointment to meet the researcher and for baseline anthropomorphic measures and digital photographs to be acquired. These parameters were inputted into the avatar creation programme while participants completed three questionnaires: (i) Impact of Weight on Quality of Life-lite (IWQOL-lite) [39]; (ii) weight efficacy lifestyle-short form questionnaire (WEL-SF) [40]; and EQ-5D-5L [41] to monitor changes in wellbeing during the study period and identify the best tool to be adopted in a future RCT if warranted".

6a-ii) Describe whether and how “use” (including intensity of use/dosage) was defined/measured/monitored

Describe whether and how “use” (including intensity of use/dosage) was defined/measured/monitored (logins, logfile analysis, etc.). Use/adoption metrics are important process outcomes that should be reported in any ehealth trial.

	1	2	3	4	5	
subitem not at all important	○	○	◉	○	○	essential

Clear selection

Your response is too large. Try shortening some answers.

Does your paper address subitem 6a-ii?

Copy and paste relevant sections from manuscript text

The feasibility paper addresses subitem 6a-ii as follows in the intervention section: "Once the avatar was generated (less than 5 minutes post data input), the participant was introduced to their avatar and programme operation and given a unique username and password to allow them to access their avatar online via computer or tablet. Participants were encouraged to explore how increasing/decreasing weight impacts on avatar appearance, consider 5% and 10% weight loss goals promoted by the weight management routine care programme alongside the BMI boundary health risks as their avatar increased and decreased in weight.

Follow-up appointments with the researcher coincided with weight management service routine care appointments at 1-month (T1) (immediate effect) and 3-month (T2) (programme completion short term effect). A further appointment to meet with the researcher was planned for 6 months (T3) (3-month post-programme completion - moderate term progress). At each of these appointments the researcher collected completed surveys and inputted any change in weight into the avatar programme. The changes in avatar appearance were presented and discussed with the participants at each researcher appointment and participants were encouraged to use the avatar to visualise progress".

6a-iii) Describe whether, how, and when qualitative feedback from participants was obtained

Describe whether, how, and when qualitative feedback from participants was obtained (e.g., through emails, feedback forms, interviews, focus groups).

	1	2	3	4	5	
subitem not at all important	○	○	○	○	◉	essential

Clear selection

Your response is too large. Try shortening some answers.

Does your paper address subitem 6a-iii?

Copy and paste relevant sections from manuscript text

The paper addresses subitem 6a-iii in the methods section as follows under the recruitment section: "A sub-sample of participants within both arms who completed the 12-week weight management programme were invited to take part in individual interviews, 3-6 months post intervention commencement and share their experiences of using the avatar programme.

Healthcare professionals involved in delivering the weight loss programme and who had experience of using the avatar programme, were invited to participate in a semi-structured interview at the conclusion of the study data collection phase. This was to explore their experiences of using the avatar programme as an adjunct to routine care".

6b) Any changes to trial outcomes after the trial commenced, with reasons

Does your paper address CONSORT subitem 6b? *

Copy and paste relevant sections from the manuscript (include quotes in quotation marks "like this" to indicate direct quotes from your manuscript), or elaborate on this item by providing additional information not in the ms, or briefly explain why the item is not applicable/relevant for your study

No changes to trial outcomes were made.

7a) How sample size was determined

NPT: When applicable, details of whether and how the clustering by care provides or centers was addressed

Your response is too large. Try shortening some answers.

7a-i) Describe whether and how expected attrition was taken into account when calculating the sample size

Describe whether and how expected attrition was taken into account when calculating the sample size.

	1	2	3	4	5	
subitem not at all important	○	○	○	○	◉	essential

Clear selection

Does your paper address subitem 7a-i?

Copy and paste relevant sections from manuscript title (include quotes in quotation marks "like this" to indicate direct quotes from your manuscript), or elaborate on this item by providing additional information not in the ms, or briefly explain why the item is not applicable/relevant for your study

The feasibility paper addresses subitem 7a-i as follows under sample size: "The target for sample size for the feasibility study trial was between 30-60 patients overall [42]. The sample size for interviews with service users and healthcare professional was based on data saturation with 10-16 interviews anticipated [43]".

7b) When applicable, explanation of any interim analyses and stopping guidelines

Does your paper address CONSORT subitem 7b? *

Copy and paste relevant sections from the manuscript (include quotes in quotation marks "like this" to indicate direct quotes from your manuscript), or elaborate on this item by providing additional information not in the ms, or briefly explain why the item is not applicable/relevant for your study

Not applicable as interim analyses were not undertaken; neither were there any stopping guidleines.

8a) Method used to generate the random allocation sequence

[illegible]

Your response is too large. Try shortening some answers.

Does your paper address CONSORT subitem 8a? *

Copy and paste relevant sections from the manuscript (include quotes in quotation marks "like this" to indicate direct quotes from your manuscript), or elaborate on this item by providing additional information not in the ms, or briefly explain why the item is not applicable/relevant for your study

The paper addresses CONSORT subitem 8a. This has been outlined in the recruitment section as follows: "A cluster randomisation approach was adopted to allocate patients to routine care or routine care plus avatar based on geographical location of weight management service intervention".

8b) Type of randomisation; details of any restriction (such as blocking and block size)

Does your paper address CONSORT subitem 8b? *

Copy and paste relevant sections from the manuscript (include quotes in quotation marks "like this" to indicate direct quotes from your manuscript), or elaborate on this item by providing additional information not in the ms, or briefly explain why the item is not applicable/relevant for your study

The paper addresses CONSORT subitem 8b. This has been outlined in the recruitment section as follows: "A cluster randomisation approach was adopted to allocate patients to routine care or routine care plus avatar based on geographical location of weight management service intervention".

9) Mechanism used to implement the random allocation sequence (such as sequentially numbered containers), describing any steps taken to conceal the sequence until interventions were assigned

Your response is too large. Try shortening some answers.

Does your paper address CONSORT subitem 9? *

Copy and paste relevant sections from the manuscript (include quotes in quotation marks "like this" to indicate direct quotes from your manuscript), or elaborate on this item by providing additional information not in the ms, or briefly explain why the item is not applicable/relevant for your study

The paper addresses CONSORT subitem 9. This has been outlined in the recruitment section as follows: "A cluster randomisation approach was adopted to allocate patients to routine care or routine care plus avatar based on geographical location of weight management service intervention".

10) Who generated the random allocation sequence, who enrolled participants, and who assigned participants to interventions

Does your paper address CONSORT subitem 10? *

Copy and paste relevant sections from the manuscript (include quotes in quotation marks "like this" to indicate direct quotes from your manuscript), or elaborate on this item by providing additional information not in the ms, or briefly explain why the item is not applicable/relevant for your study

The paper does not specifically address CONSORT subitem 10 as a random allocation sequence was not used but a cluster randomisation approach was adopted by the rfesewarch team.

11a) If done, who was blinded after assignment to interventions (for example, participants, care providers, those assessing outcomes) and how

NPT: Whether or not administering co-interventions were blinded to group assignment

Your response is too large. Try shortening some answers.

11a-i) Specify who was blinded, and who wasn't

Specify who was blinded, and who wasn't. Usually, in web-based trials it is not possible to blind the participants [1, 3] (this should be clearly acknowledged), but it may be possible to blind outcome assessors, those doing data analysis or those administering co-interventions (if any).

	1	2	3	4	5	
subitem not at all important	◉	○	○	○	○	essential

Clear selection

Does your paper address subitem 11a-i? *

Copy and paste relevant sections from the manuscript (include quotes in quotation marks "like this" to indicate direct quotes from your manuscript), or elaborate on this item by providing additional information not in the ms, or briefly explain why the item is not applicable/relevant for your study

The paper does not address subitem 11a-i as blinding was not possible.

11a-ii) Discuss e.g., whether participants knew which intervention was the "intervention of interest" and which one was the "comparator"

Informed consent procedures (4a-ii) can create biases and certain expectations - discuss e.g., whether participants knew which intervention was the "intervention of interest" and which one was the "comparator".

	1	2	3	4	5	
subitem not at all important	○	○	○	◉	○	essential

Clear selection

Your response is too large. Try shortening some answers.

Does your paper address subitem 11a-ii?

Copy and paste relevant sections from the manuscript (include quotes in quotation marks "like this" to indicate direct quotes from your manuscript), or elaborate on this item by providing additional information not in the ms, or briefly explain why the item is not applicable/relevant for your study

"However, while routine care participants underwent the same process of meeting the researcher and replicating activity of the intervention, visualisation of, and access to, their avatar was withheld until the final weight management service appointment (12-week)".

11b) If relevant, description of the similarity of interventions

(this item is usually not relevant for ehealth trials as it refers to similarity of a placebo or sham intervention to a active medication/intervention)

Does your paper address CONSORT subitem 11b? *

Copy and paste relevant sections from the manuscript (include quotes in quotation marks "like this" to indicate direct quotes from your manuscript), or elaborate on this item by providing additional information not in the ms, or briefly explain why the item is not applicable/relevant for your study

The paper does not address CONSORT subitem 11b as there was only one intervention used.

12a) Statistical methods used to compare groups for primary and secondary outcomes

NPT: When applicable, details of whether and how the clustering by care providers or centers was addressed

Does your paper address CONSORT subitem 12a? *

Copy and paste relevant sections from the manuscript (include quotes in quotation marks "like this" to indicate direct quotes from your manuscript), or elaborate on this item by providing additional information not in the ms, or briefly explain why the item is not applicable/relevant for your study

The paper addresses CONSORT subitem 12a

Your response is too large. Try shortening some answers.

12a-i) Imputation techniques to deal with attrition / missing values

Imputation techniques to deal with attrition / missing values: Not all participants will use the intervention/comparator as intended and attrition is typically high in ehealth trials. Specify how participants who did not use the application or dropped out from the trial were treated in the statistical analysis (a complete case analysis is strongly discouraged, and simple imputation techniques such as LOCF may also be problematic [4]).

	1	2	3	4	5	
subitem not at all important	○	○	◉	○	○	essential

Clear selection

Does your paper address subitem 12a-i? *

Copy and paste relevant sections from the manuscript (include quotes in quotation marks "like this" to indicate direct quotes from your manuscript), or elaborate on this item by providing additional information not in the ms, or briefly explain why the item is not applicable/relevant for your study

The paper address subitem 12a-i

12b) Methods for additional analyses, such as subgroup analyses and adjusted analyses

Does your paper address CONSORT subitem 12b? *

Copy and paste relevant sections from the manuscript (include quotes in quotation marks "like this" to indicate direct quotes from your manuscript), or elaborate on this item by providing additional information not in the ms, or briefly explain why the item is not applicable/relevant for your study

Not applicable as no additional analyses, such as subgroup analyses were undertaken.

X26) REB/IRB Approval and Ethical Considerations [recommended as subheading under "Methods"] (not a CONSORT item)

Your response is too large. Try shortening some answers.

X26-i) Comment on ethics committee approval

	1	2	3	4	5	
subitem not at all important	○	○	○	○	◉	essential

Clear selection

Does your paper address subitem X26-i?

Copy and paste relevant sections from the manuscript (include quotes in quotation marks "like this" to indicate direct quotes from your manuscript), or elaborate on this item by providing additional information not in the ms, or briefly explain why the item is not applicable/relevant for your study

The paper address subitem X26-i and has a Ethical Considerations as a subheading under "Methods".

x26-ii) Outline informed consent procedures

Outline informed consent procedures e.g., if consent was obtained offline or online (how? Checkbox, etc.?), and what information was provided (see 4a-ii). See [6] for some items to be included in informed consent documents.

	1	2	3	4	5	
subitem not at all important	○	○	○	○	◉	essential

Clear selection

Your response is too large. Try shortening some answers.

Does your paper address subitem X26-ii?

Copy and paste relevant sections from the manuscript (include quotes in quotation marks "like this" to indicate direct quotes from your manuscript), or elaborate on this item by providing additional information not in the ms, or briefly explain why the item is not applicable/relevant for your study

The paper addresses subitem X26-ii under the recruitment subheading of the methods section as follows: "Potential participants were identified by the weight management service clinical team (Mid Yorkshire Hospitals NHS Trust) following initial assessment of suitability to participate in a weight management programme and study participation screening. Potential participants were given an information pack, including invitation letter, information sheet, researcher permission to contact form and postage paid envelope to read, complete and return after the appointment. The forms were returned to the weight management service and a secure database of potential participants created by the NHS research administrator assigned to this project. The researcher confirmed with the administrator on a weekly basis those patients who had returned the permission to contact forms and telephoned those patients interested in participating to describe the study further and confirm willingness to participate. A cluster randomisation approach was adopted to allocate patients to routine care or routine care plus avatar based on geographical location of weight management service intervention".

X26-iii) Safety and security procedures

Safety and security procedures, incl. privacy considerations, and any steps taken to reduce the likelihood or detection of harm (e.g., education and training, availability of a hotline)

	1	2	3	4	5	
subitem not at all important	○	○	○	○	●	essential

Clear selection

Does your paper address subitem X26-iii?

Copy and paste relevant sections from the manuscript (include quotes in quotation marks "like this" to indicate direct quotes from your manuscript), or elaborate on this item by providing additional information not in the ms, or briefly explain why the item is not applicable/relevant for your study

The paper addresses subitem X26-iii in regards to safety procedures in the ethical consideraation section as follows"...a process of medical referral and reporting of any potential harm as a result of the study participation established"

Your response is too large. Try shortening some answers.

RESULTS

13a) For each group, the numbers of participants who were randomly assigned, received intended treatment, and were analysed for the primary outcome
NPT: The number of care providers or centers performing the intervention in each group and the number of patients treated by each care provider in each center

Does your paper address CONSORT subitem 13a? *

Copy and paste relevant sections from the manuscript (include quotes in quotation marks "like this" to indicate direct quotes from your manuscript), or elaborate on this item by providing additional information not in the ms, or briefly explain why the item is not applicable/relevant for your study

The paper addresses CONSORT subitem 13a as follows: "Recruitment took place between March and December 2019. During this time, 51 potential participants were identified as being suitable for participation in the study and information provided. Of these, five (n=5/51; 9.8%) declined participation and three did not attend the initial appointment and were discharged from the weight management service (see Figure 1)".

13b) For each group, losses and exclusions after randomisation, together with reasons

Does your paper address CONSORT subitem 13b? (NOTE: Preferably, this is shown in a CONSORT flow diagram) *

Copy and paste relevant sections from the manuscript (include quotes in quotation marks "like this" to indicate direct quotes from your manuscript), or elaborate on this item by providing additional information not in the ms, or briefly explain why the item is not applicable/relevant for your study

The paper address CONSORT subitem 13b. A CONSORT flow diagram is provided.

Your response is too large. Try shortening some answers.

13b-i) Attrition diagram

Strongly recommended: An attrition diagram (e.g., proportion of participants still logging in or using the intervention/comparator in each group plotted over time, similar to a survival curve) or other figures or tables demonstrating usage/dose/engagement.

	1	2	3	4	5	
subitem not at all important	◉	○	○	○	○	essential

Clear selection

Does your paper address subitem 13b-i?

Copy and paste relevant sections from the manuscript or cite the figure number if applicable (include quotes in quotation marks "like this" to indicate direct quotes from your manuscript), or elaborate on this item by providing additional information not in the ms, or briefly explain why the item is not applicable/relevant for your study

The paper does not address subitem 13b-i. We have not prioivided a specific Attrition diagram but this is reported inb the Participation flow Chart - Figure 1.

14a) Dates defining the periods of recruitment and follow-up

Does your paper address CONSORT subitem 14a? *

Copy and paste relevant sections from the manuscript (include quotes in quotation marks "like this" to indicate direct quotes from your manuscript), or elaborate on this item by providing additional information not in the ms, or briefly explain why the item is not applicable/relevant for your study

The paper address CONSORT subitem 14a as follows "Recruitment took place between March and December 2019" and in the eintervenmtion section of the methods "Follow-up appointments with the researcher coincided with weight management service routine care appointments at 1-month (T1) (immediate effect) and 3-month (T2) (programme completion short term effect)".

Your response is too large. Try shortening some answers.

14a-i) Indicate if critical “secular events” fell into the study period

Indicate if critical “secular events” fell into the study period, e.g., significant changes in Internet resources available or “changes in computer hardware or Internet delivery resources”

	1	2	3	4	5	
subitem not at all important	(selected)					essential

Clear selection

Does your paper address subitem 14a-i?

Copy and paste relevant sections from the manuscript (include quotes in quotation marks "like this" to indicate direct quotes from your manuscript), or elaborate on this item by providing additional information not in the ms, or briefly explain why the item is not applicable/relevant for your study

There were no critical “secular events” during the study period.

14b) Why the trial ended or was stopped (early)

Does your paper address CONSORT subitem 14b? *

Copy and paste relevant sections from the manuscript (include quotes in quotation marks "like this" to indicate direct quotes from your manuscript), or elaborate on this item by providing additional information not in the ms, or briefly explain why the item is not applicable/relevant for your study

The feasibility study was not stoipped early.

15) A table showing baseline demographic and clinical characteristics for each group

NPT: When applicable, a description of care providers (case volume, qualification, expertise, etc.) and centers (volume) in each group

Your response is too large. Try shortening some answers.

Does your paper address CONSORT subitem 15? *

Copy and paste relevant sections from the manuscript (include quotes in quotation marks "like this" to indicate direct quotes from your manuscript), or elaborate on this item by providing additional information not in the ms, or briefly explain why the item is not applicable/relevant for your study

The paper addresses CONSORT subitem 15 as follows": "Baseline data were collected for 43 participants. A total of 10 males were recruited into the study (7 into the routine care plus avatar arm and 3 into the routine care arm). On commencing the programme, their mean age was 50yrs 10 months (range 39-63yrs) and mean weight 140.2kg (range 114.6-173.2 kg). A total of 33 females were enrolled into the study at T0 (23 into the intervention arm and 10 into the routine care arm). On commencing the programme, their mean age was 40yrs 1 month (range 21-57yrs) and mean weight 120.5kg (range 89.6-156.4 kg). Mean BMI was 43.3 (range 38.6 - 49.5).

The initial mean weight of participants was greater in the intervention arm than routine care arm (126.3 kg vs 122.9 kg) but the pattern of weight loss was similar across both arms of the study in period T0 – T1. During T1-T2 weight loss accelerated in the intervention arm suggesting that access to the self-resembling avatar may promote greater engagement with weight loss initiatives in the short to medium term (Figure 2).
The mean change in weight of the participants from T0 to T2 was 4.5kg (95% CI: 2.7-6.3) in routine care arm and 5.3kg (95% CI: 3.9-6.8) in the intervention arm. The initial mean BMI of participants was greater in the intervention arm than routine care arm (44.0 vs 42.4 kg). However, reflecting the greater weight loss in the intervention arm, the mean BMI of participants in both groups was identical at T2 (BMI 41.1).
Quality-of-life and self-efficacy showed improvements in participants scores across both arms of the study, but greater improvement was noted within the intervention arm (Figure 3). Mean initial scores were comparable across the arms (107.9 routine care; 109.5 intervention arm) but participants in the intervention arm reported greater improvement at both T1 (105.5 routine care; 99.7 intervention arm) and T2 (100.1 routine care; 81.2 intervention arm). Improvement in participant self-assessment of health at time of appointment was also noted over the study time frame for participants in both arms of the study using EQ-5D-5L VAS score although once again, greater improvement was noted within the intervention arm. Mean VAS scores for the routine arm were 49.1 (T0), 50.2 (T1) and 52.8 (T2) whereas for the intervention arm, mean VAS scores were 43.6 (T0), 58.7 (T1), 62.5 (T2).
Having access to and visualizing change in the self-resembling avatar improved weight loss motivation and perception of self-well-being. In contrast, the weight efficacy lifestyle questionnaire identified greater improvements in participant self-belief to control eating behaviours in the routine care arm (Figure 4) with a mean improvement in score between T0 and T2 of 17.6 in the routine arm and 12.1 in the intervention arm. It is unclear why the results from this questionnaire contrast the findings of the other assessments. This may reflect the wording of statements, purpose of questionnaire or interpretation by participants. Regardless, it is important to note that use of a single questionnaire alone will not allow for understanding of the complex motivations, enablers and barriers to weight loss accelerated in the intervention arm suggesting that access to the self-resembling avatar may promote greater engagement with weight loss initiatives in the short to medium term (Figure 2)".

Your response is too large. Try shortening some answers.

15-i) Report demographics associated with digital divide issues

In ehealth trials it is particularly important to report demographics associated with digital divide issues, such as age, education, gender, social-economic status, computer/Internet/ehealth literacy of the participants, if known.

	1	2	3	4	5	
subitem not at all important	●	○	○	○	○	essential

Clear selection

Does your paper address subitem 15-i? *

Copy and paste relevant sections from the manuscript (include quotes in quotation marks "like this" to indicate direct quotes from your manuscript), or elaborate on this item by providing additional information not in the ms, or briefly explain why the item is not applicable/relevant for your study

The paper does not address subitem 15-i. We did not collect data on demographics associated with digital divide issues. Participants who were recruited had mobile phones and/or tablets to access the avatar online.

16) For each group, number of participants (denominator) included in each analysis and whether the analysis was by original assigned groups

16-i) Report multiple “denominators” and provide definitions

Report multiple “denominators” and provide definitions: Report N’s (and effect sizes) “across a range of study participation [and use] thresholds” [1], e.g., N exposed, N consented, N used more than x times, N used more than y weeks, N participants “used” the intervention/comparator at specific pre-defined time points of interest (in absolute and relative numbers per group). Always clearly define “use” of the intervention.

	1	2	3	4	5	
subitem not at all important	○	○	○	●	○	essential

Your response is too large. Try shortening some answers.

Does your paper address subitem 16-i? *

Copy and paste relevant sections from the manuscript (include quotes in quotation marks "like this" to indicate direct quotes from your manuscript), or elaborate on this item by providing additional information not in the ms, or briefly explain why the item is not applicable/relevant for your study

The paper addresses subitem 16-i as follows: "The initial mean weight of participants was greater in the intervention arm than routine care arm (126.3 kg vs 122.9 kg) but the pattern of weight loss was similar across both arms of the study in period T0 – T1. During T1-T2 weight loss accelerated in the intervention arm suggesting that access to the self-resembling avatar may promote greater engagement with weight loss initiatives in the short to medium term (Figure 2).
The mean change in weight of the participants from T0 to T2 was 4.5kg (95% CI: 2.7-6.3) in routine care arm and 5.3kg (95% CI: 3.9-6.8) in the intervention arm. The initial mean BMI of participants was greater in the intervention arm than routine care arm (44.0 vs 42.4 kg). However, reflecting the greater weight loss in the intervention arm, the mean BMI of participants in both groups was identical at T2 (BMI 41.1).
Quality-of-life and self-efficacy showed improvements in participants scores across both arms of the study, but greater improvement was noted within the intervention arm (Figure 3). Mean initial scores were comparable across the arms (107.9 routine care; 109.5 intervention arm) but participants in the intervention arm reported greater improvement at both T1 (105.5 routine care; 99.7 intervention arm) and T2 (100.1 routine care; 81.2 intervention arm). Improvement in participant self-assessment of health at time of appointment was also noted over the study time frame for participants in both arms of the study using EQ-5D-5L VAS score although once again, greater improvement was noted within the intervention arm. Mean VAS scores for the routine arm were 49.1 (T0), 50.2 (T1) and 52.8 (T2) whereas for the intervention arm, mean VAS scores were 43.6 (T0), 58.7 (T1), 62.5 (T2).
Having access to and visualizing change in the self-resembling avatar improved weight loss motivation and perception of self-well-being. In contrast, the weight efficacy lifestyle questionnaire identified greater improvements in participant self-belief to control eating behaviours in the routine care arm (Figure 4) with a mean improvement in score between T0 and T2 of 17.6 in the routine arm and 12.1 in the intervention arm. It is unclear why the results from this questionnaire contrast the findings of the other assessments. This may reflect the wording of statements, purpose of questionnaire or interpretation by participants. Regardless, it is important to note that use of a single questionnaire alone will not allow for understanding of the complex motivations, enablers and barriers to weight loss".

Your response is too large. Try shortening some answers.

16-ii) Primary analysis should be intent-to-treat

Primary analysis should be intent-to-treat, secondary analyses could include comparing only “users”, with the appropriate caveats that this is no longer a randomized sample (see 18-i).

	1	2	3	4	5	
subitem not at all important	◉	○	○	○	○	essential

Clear selection

Does your paper address subitem 16-ii?

Copy and paste relevant sections from the manuscript (include quotes in quotation marks "like this" to indicate direct quotes from your manuscript), or elaborate on this item by providing additional information not in the ms, or briefly explain why the item is not applicable/relevant for your study

The paper does not address subitem 16-ii. This was a feasbility study; intent-to-treat was not applicable.

17a) For each primary and secondary outcome, results for each group, and the estimated effect size and its precision (such as 95% confidence interval)

Does your paper address CONSORT subitem 17a? *

Copy and paste relevant sections from the manuscript (include quotes in quotation marks "like this" to indicate direct quotes from your manuscript), or elaborate on this item by providing additional information not in the ms, or briefly explain why the item is not applicable/relevant for your study

This was a feasibility study, the study was not powered to assess the effectiveness of the intervention.

Your response is too large. Try shortening some answers.

17a-i) Presentation of process outcomes such as metrics of use and intensity of use

In addition to primary/secondary (clinical) outcomes, the presentation of process outcomes such as metrics of use and intensity of use (dose, exposure) and their operational definitions is critical. This does not only refer to metrics of attrition (13-b) (often a binary variable), but also to more continuous exposure metrics such as “average session length”. These must be accompanied by a technical description how a metric like a “session” is defined (e.g., timeout after idle time) [1] (report under item 6a).

	1	2	3	4	5	
subitem not at all important	◉	○	○	○	○	essential

Clear selection

Your response is too large. Try shortening some answers.

Does your paper address subitem 17a-i?

Copy and paste relevant sections from the manuscript (include quotes in quotation marks "like this" to indicate direct quotes from your manuscript), or elaborate on this item by providing additional information not in the ms, or briefly explain why the item is not applicable/relevant for your study

The paper does not address subitem 17a-i as we did not collect data on process outcomes such as metrics of use and intensity of use. However, we report the pricipal findgings as follows: "The aim of this study was to investigate the feasibility, acceptability and implementation of a randomised design to determine the case for future development and evaluation of avatar-based technology in a future randomised controlled trial.
Following the recommendations for feasibility studies [45], feasibility was assessed through examining recruitment and retention (attrition) rates, together with participant's and healthcare professionals' experiences of the use of avatar-based technology as an adjunct to a weight management programme. In addition, we reported pre-post intervention effect sizes on primary (weight loss) and other outcomes.
Recruitment was found to be feasible with over half of people consenting to participate and the target of recruiting at least 30 into the intervention arm during the 6-month recruitment period was met. Reasons for declining to participate in the study were disinterest in the research and lack of guarantee of being in the intervention arm. Three recruited participants failed to attend their initial appointment and were discharged from the weight management service. The numbers recruited to the intervention arm (n = 30) suggest that a personalised avatar, as an adjunct to a weight management programme, would be feasible to offer and would be of interest to participants. However, attrition from the programme was relatively high (n=19/46; 41.3%).
In terms of acceptability all study participants mentioned experiencing benefits from using the avatar-based technology. Most participants emphasised the importance of avatar appearance as a reality check, whilst healthcare professionals felt it was important for patients to visualise 'reality' and confront denial.
Most participants in our study also reported added psychological benefits, such as increased motivation to lose weight, improved self-esteem and self-confidence.
Finally, although the feasibility study was not powered to detect a difference in weight, the study found a 10% greater mean weight loss with routine care plus avatar compared with routine care weight management programme".

17b) For binary outcomes, presentation of both absolute and relative effect sizes is recommended

Your response is too large. Try shortening some answers.

Does your paper address CONSORT subitem 17b? *

Copy and paste relevant sections from the manuscript (include quotes in quotation marks "like this" to indicate direct quotes from your manuscript), or elaborate on this item by providing additional information not in the ms, or briefly explain why the item is not applicable/relevant for your study

This was a feasibility study, the study was not powered to assess both absolute and relative effect sizes. However, we report the pricicpal findings: "The aim of this study was to investigate the feasibility, acceptability and implementation of a randomised design to determine the case for future development and evaluation of avatar-based technology in a future randomised controlled trial.

Following the recommendations for feasibility studies [45], feasibility was assessed through examining recruitment and retention (attrition) rates, together with participant's and healthcare professionals' experiences of the use of avatar-based technology as an adjunct to a weight management programme. In addition, we reported pre-post intervention effect sizes on primary (weight loss) and other outcomes.
Recruitment was found to be feasible with over half of people consenting to participate and the target of recruiting at least 30 into the intervention arm during the 6-month recruitment period was met. Reasons for declining to participate in the study were disinterest in the research and lack of guarantee of being in the intervention arm. Three recruited participants failed to attend their initial appointment and were discharged from the weight management service. The numbers recruited to the intervention arm (n = 30) suggest that a personalised avatar, as an adjunct to a weight management programme, would be feasible to offer and would be of interest to participants. However, attrition from the programme was relatively high (n=19/46; 41.3%).
In terms of acceptability all study participants mentioned experiencing benefits from using the avatar-based technology. Most participants emphasised the importance of avatar appearance as a reality check, whilst healthcare professionals felt it was important for patients to visualise 'reality' and confront denial.
Most participants in our study also reported added psychological benefits, such as increased motivation to lose weight, improved self-esteem and self-confidence.
Finally, although the feasibility study was not powered to detect a difference in weight, the study found a 10% greater mean weight loss with routine care plus avatar compared with routine care weight management programme".

18) Results of any other analyses performed, including subgroup analyses and adjusted analyses, distinguishing pre-specified from exploratory

Your response is too large. Try shortening some answers.

Does your paper address CONSORT subitem 18? *

Copy and paste relevant sections from the manuscript (include quotes in quotation marks "like this" to indicate direct quotes from your manuscript), or elaborate on this item by providing additional information not in the ms, or briefly explain why the item is not applicable/relevant for your study

The paper does not address CONSORT subitem 18 as no other asubgroup analyses and adjusted analyses, distinguishing pre-specified from exploratory were undertaken.

18-i) Subgroup analysis of comparing only users

A subgroup analysis of comparing only users is not uncommon in ehealth trials, but if done, it must be stressed that this is a self-selected sample and no longer an unbiased sample from a randomized trial (see 16-iii).

	1	2	3	4	5	
subitem not at all important	◉	○	○	○	○	essential

Clear selection

Does your paper address subitem 18-i?

Copy and paste relevant sections from the manuscript (include quotes in quotation marks "like this" to indicate direct quotes from your manuscript), or elaborate on this item by providing additional information not in the ms, or briefly explain why the item is not applicable/relevant for your study

The paper does not address subitem 18-i as subgroup analysis of comparing only users was not undertaken.

19) All important harms or unintended effects in each group

(for specific guidance see CONSORT for harms)

Your response is too large. Try shortening some answers.

Does your paper address CONSORT subitem 19? *

Copy and paste relevant sections from the manuscript (include quotes in quotation marks "like this" to indicate direct quotes from your manuscript), or elaborate on this item by providing additional information not in the ms, or briefly explain why the item is not applicable/relevant for your study

The paper addresses CONSORT subitem 19. Only potential negative consquence was found in the qualitative data as follows: "However, a few participants found initial viewing of the avatar uncomfortable, but reported that the appearance of their avatar was a 'reality check' of their current selves which provided the added motivation to lose weight:

'... it was all a bit shocking to be fair [seeing avatar for 1st time]...I don't look at myself very much in the mirror...' (MM47: Male).

'It gives you an objective look on how others would see you' (MF13: Female).

'Devastated - that I had let myself get like that so... yeah... embarrassed...it spurred me on to get rid of all that round my belly...I know...as I get thinner I will get fitter and get rid of this lung issue and then I will get to where I want to be and I will go to the gym more and even do more at home. I will definitely walk the dog more; I'm trying to walk the dog faster so I am exerting myself more...so yeah, seeing that end result is brilliant to [compare with] where I was' (MF23: Female)."
Also, in the discussion: "In terms of acceptability all study participants mentioned experiencing benefits from using the avatar-based technology. Most participants emphasised the importance of avatar appearance as a reality check, whilst healthcare professionals felt it was important for patients to visualise 'reality' and confront denial".

19-i) Include privacy breaches, technical problems

Include privacy breaches, technical problems. This does not only include physical "harm" to participants, but also incidents such as perceived or real privacy breaches [1], technical problems, and other unexpected/unintended incidents. "Unintended effects" also includes unintended positive effects [2].

	1	2	3	4	5	
subitem not at all important	(•)	○	○	○	○	essential

Clear selection

Your response is too large. Try shortening some answers.

Does your paper address subitem 19-i?

Copy and paste relevant sections from the manuscript (include quotes in quotation marks "like this" to indicate direct quotes from your manuscript), or elaborate on this item by providing additional information not in the ms, or briefly explain why the item is not applicable/relevant for your study

No privacy breaches occured.

19-ii) Include qualitative feedback from participants or observations from staff/researchers

Include qualitative feedback from participants or observations from staff/researchers, if available, on strengths and shortcomings of the application, especially if they point to unintended/unexpected effects or uses. This includes (if available) reasons for why people did or did not use the application as intended by the developers.

	1	2	3	4	5	
subitem not at all important	○	○	○	○	◉	essential

Clear selection

Your response is too large. Try shortening some answers.

Does your paper address subitem 19-ii?

Copy and paste relevant sections from the manuscript (include quotes in quotation marks "like this" to indicate direct quotes from your manuscript), or elaborate on this item by providing additional information not in the ms, or briefly explain why the item is not applicable/relevant for your study

The paper address subitem 19-ii as follows in the results section: "Thirteen participants (11F, 2M) and two HCPs volunteered to be interviewed about their experiences of using the routine care plus avatar weight loss programme. In terms of acceptability, most participants found using the personalised avatar an acceptable and positive experience" and "Both healthcare practitioners were dieticians and/or nutritionists and both felt that the personalised avatar, as an adjunct to a weight management programme, was a useful addition to the questionnaires available:

'I think it exceeded my expectations…because obviously she takes a photo of the patient and then it's got their face on it and obviously their body…I know it didn't have any clothing or anything like that [underwear only], but I don't think that really matters because they get to see the actual body without the clothing… I think that's better because they can see their skin and where they are actually carrying the weight… I think overall it has been beneficial to have it as part of the programme' (HCP1, female)".

DISCUSSION

22) Interpretation consistent with results, balancing benefits and harms, and considering other relevant evidence

NPT: In addition, take into account the choice of the comparator, lack of or partial blinding, and unequal expertise of care providers or centers in each group

Your response is too large. Try shortening some answers.

22-i) Restate study questions and summarize the answers suggested by the data, starting with primary outcomes and process outcomes (use)

Restate study questions and summarize the answers suggested by the data, starting with primary outcomes and process outcomes (use).

	1	2	3	4	5	
subitem not at all important	○	○	○	○	◉	essential

Clear selection

Your response is too large. Try shortening some answers.

Does your paper address subitem 22-i? *

Copy and paste relevant sections from the manuscript (include quotes in quotation marks "like this" to indicate direct quotes from your manuscript), or elaborate on this item by providing additional information not in the ms, or briefly explain why the item is not applicable/relevant for your study

paper address subitem 22-i. The principal findings are summarised as follows: "The aim of this study was to investigate the feasibility, acceptability and implementation of a randomised design to determine the case for future development and evaluation of avatar-based technology in a future randomised controlled trial.

Following the recommendations for feasibility studies [45], feasibility was assessed through examining recruitment and retention (attrition) rates, together with participant's and healthcare professionals' experiences of the use of avatar-based technology as an adjunct to a weight management programme. In addition, we reported pre-post intervention effect sizes on primary (weight loss) and other outcomes.

Recruitment was found to be feasible with over half of people consenting to participate and the target of recruiting at least 30 into the intervention arm during the 6-month recruitment period was met. Reasons for declining to participate in the study were disinterest in the research and lack of guarantee of being in the intervention arm. Three recruited participants failed to attend their initial appointment and were discharged from the weight management service. The numbers recruited to the intervention arm (n = 30) suggest that a personalised avatar, as an adjunct to a weight management programme, would be feasible to offer and would be of interest to participants. However, attrition from the programme was relatively high (n=19/46; 41.3%).

In terms of acceptability all study participants mentioned experiencing benefits from using the avatar-based technology. Most participants emphasised the importance of avatar appearance as a reality check, whilst healthcare professionals felt it was important for patients to visualise 'reality' and confront denial.

Most participants in our study also reported added psychological benefits, such as increased motivation to lose weight, improved self-esteem and self-confidence.
Finally, although the feasibility study was not powered to detect a difference in weight, the study found a 10% greater mean weight loss with routine care plus avatar compared with routine care weight management programme."

Your response is too large. Try shortening some answers.

22-ii) Highlight unanswered new questions, suggest future research

Highlight unanswered new questions, suggest future research.

	1	2	3	4	5	
subitem not at all important	○	○	○	○	◉	essential

Clear selection

Does your paper address subitem 22-ii?

Copy and paste relevant sections from the manuscript (include quotes in quotation marks "like this" to indicate direct quotes from your manuscript), or elaborate on this item by providing additional information not in the ms, or briefly explain why the item is not applicable/relevant for your study

The paper addresses subitem 22-ii as follows in the limitations sections : "As this was a feasibility study, assessing the effectiveness of the intervention was not the primary aim. However, future research should examine effectiveness of the avatar within an RCT design as well as better understand the economic impact."

20) Trial limitations, addressing sources of potential bias, imprecision, and, if relevant, multiplicity of analyses

20-i) Typical limitations in ehealth trials

Typical limitations in ehealth trials: Participants in ehealth trials are rarely blinded. Ehealth trials often look at a multiplicity of outcomes, increasing risk for a Type I error. Discuss biases due to non-use of the intervention/usability issues, biases through informed consent procedures, unexpected events.

	1	2	3	4	5	
subitem not at all important	○	○	○	○	◉	essential

Clear selection

Your response is too large. Try shortening some answers.

Does your paper address subitem 20-i? *

Copy and paste relevant sections from the manuscript (include quotes in quotation marks "like this" to indicate direct quotes from your manuscript), or elaborate on this item by providing additional information not in the ms, or briefly explain why the item is not applicable/relevant for your study

The paper addresses subitem 20-i as follows under the limitations secction: "Limitations to this study include the small sample size and relatively high attrition. However, it still remains one of the larger sample size studies reported to date. Despite these limitations, this feasibility study has illustrated that avatar-based technology may successfully promote engagement with and motivation to lose weight as part of a weight management programme.

This feasibility study demonstrates the possibilities of using avatar-based technology to motivate engagement with a weight management programme in the short term. The findings also suggest that avatar-based technology may support greater self-confidence, belief and efficacy in weight loss ambitions."

21) Generalisability (external validity, applicability) of the trial findings

NPT: External validity of the trial findings according to the intervention, comparators, patients, and care providers or centers involved in the trial

21-i) Generalizability to other populations

Generalizability to other populations: In particular, discuss generalizability to a general Internet population, outside of a RCT setting, and general patient population, including applicability of the study results for other organizations

	1	2	3	4	5	
subitem not at all important	(●)	( )	( )	( )	( )	essential

Clear selection

Your response is too large. Try shortening some answers.

Does your paper address subitem 21-i?

Copy and paste relevant sections from the manuscript (include quotes in quotation marks "like this" to indicate direct quotes from your manuscript), or elaborate on this item by providing additional information not in the ms, or briefly explain why the item is not applicable/relevant for your study

The paper reports the investigation of the feasibility, acceptability and implementation of a randomised design to determine the case for future development and evaluation of avatar-based technology in a randomised controlled trial. As such we were not nlooking spspcifcally at generalising to other population groups at this stage.

21-ii) Discuss if there were elements in the RCT that would be different in a routine application setting

Discuss if there were elements in the RCT that would be different in a routine application setting (e.g., prompts/reminders, more human involvement, training sessions or other co-interventions) and what impact the omission of these elements could have on use, adoption, or outcomes if the intervention is applied outside of a RCT setting.

	1	2	3	4	5	
subitem not at all important	○	◉	○	○	○	essential

Clear selection

Does your paper address subitem 21-ii?

Copy and paste relevant sections from the manuscript (include quotes in quotation marks "like this" to indicate direct quotes from your manuscript), or elaborate on this item by providing additional information not in the ms, or briefly explain why the item is not applicable/relevant for your study

The paper reports the investigation of the feasibility, acceptability and implementation of a randomised design to determine the case for future development and evaluation of avatar-based technology in a randomised controlled trial undertaken in a routine application setting.

OTHER INFORMATION

Your response is too large. Try shortening some answers.

23) Registration number and name of trial registry

Does your paper address CONSORT subitem 23? *

Copy and paste relevant sections from the manuscript (include quotes in quotation marks "like this" to indicate direct quotes from your manuscript), or elaborate on this item by providing additional information not in the ms, or briefly explain why the item is not applicable/relevant for your study

The paper addresses CONSORT subitem 23 as follows: "...registered with the International Standard Randomised Control Trial Number (17953876)."

24) Where the full trial protocol can be accessed, if available

Does your paper address CONSORT subitem 24? *

Cite a Multimedia Appendix, other reference, or copy and paste relevant sections from the manuscript (include quotes in quotation marks "like this" to indicate direct quotes from your manuscript), or elaborate on this item by providing additional information not in the ms, or briefly explain why the item is not applicable/relevant for your study

The paper addresses CONSORT subitem 24"...registered with the International Standard Randomised Control Trial Number (17953876)."

25) Sources of funding and other support (such as supply of drugs), role of funders

Your response is too large. Try shortening some answers.

Does your paper address CONSORT subitem 25? *

Copy and paste relevant sections from the manuscript (include quotes in quotation marks "like this" to indicate direct quotes from your manuscript), or elaborate on this item by providing additional information not in the ms, or briefly explain why the item is not applicable/relevant for your study

The paper addresses CONSORT subitem 25 under Acknowledgements/funding section as follows: "This study/project is funded by the National Institute for Health Research (NIHR) [RfPB programme (project reference PB-PG-1215-20016)]. The views expressed are those of the author(s) and not necessarily those of the NIHR or the Department of Health and Social Care."

X27) Conflicts of Interest (not a CONSORT item)

X27-i) State the relation of the study team towards the system being evaluated

In addition to the usual declaration of interests (financial or otherwise), also state the relation of the study team towards the system being evaluated, i.e., state if the authors/evaluators are distinct from or identical with the developers/sponsors of the intervention.

	1	2	3	4	5	
subitem not at all important	○	○	○	◉	○	essential

Clear selection

Does your paper address subitem X27-i?

Copy and paste relevant sections from the manuscript (include quotes in quotation marks "like this" to indicate direct quotes from your manuscript), or elaborate on this item by providing additional information not in the ms, or briefly explain why the item is not applicable/relevant for your study

Not specifically in the paper but on submission to the journal.

About the CONSORT EHEALTH checklist

Your response is too large. Try shortening some answers.

As a result of using this checklist, did you make changes in your manuscript? *

- ( ) yes, major changes
- (x) yes, minor changes
- ( ) no

What were the most important changes you made as a result of using this checklist?

Your answer

How much time did you spend on going through the checklist INCLUDING making changes in your manuscript *

I spent 6 hours undertaking this checklist including making changes.

As a result of using this checklist, do you think your manuscript has improved? *

- (x) yes
- ( ) no
- ( ) Other:

Your response is too large. Try shortening some answers.

Would you like to become involved in the CONSORT EHEALTH group?

This would involve for example becoming involved in participating in a workshop and writing an "Explanation and Elaboration" document

- ○ yes
- ◉ no
- ○ Other:

Clear selection

Any other comments or questions on CONSORT EHEALTH

Your answer

STOP - Save this form as PDF before you click submit

To generate a record that you filled in this form, we recommend to generate a PDF of this page (on a Mac, simply select "print" and then select "print as PDF") before you submit it.

When you submit your (revised) paper to JMIR, please upload the PDF as supplementary file.

Don't worry if some text in the textboxes is cut off, as we still have the complete information in our database. Thank you!

Final step: Click submit !

Click submit so we have your answers in our database!

Submit Clear form

Never submit passwords through Google Forms.

This content is neither created nor endorsed by Google. Report Abuse - Terms of Service - Privacy Policy

Google Forms

Your response is too large. Try shortening some answers.
